# Supplementary material for: Robust, fiducial-free drift correction for super-resolution imaging
Source: Sci Rep. 2021 Dec 8;11:23672. doi: 10.1038/s41598-021-02850-7 (PMC8655078; doi:10.1038/s41598-021-02850-7)
Supplement: Supplementary file 1 — Supplementary Information. [file 41598_2021_2850_MOESM1_ESM.pdf]

Robust, Fiducial-Free Drift Correction for Super-resolution Imaging Supplementary Information

Michael J. Wester[1,2], David J. Schodt[1], Hanieh Mazloom-Farsibaf[1,3], Mohamadreza Fazel[1,4], Sandeep Pallikkuth[1], and Keith A. Lidke[1,*]

[1]Department of Physics and Astronomy, University of New Mexico, Albuquerque, NM 87131 USA
[2]Department of Mathematics and Statistics, University of New Mexico, Albuquerque, NM 87131 USA
[3]Lyda Hill Department of Bioinformatics, UT Southwestern Medical Center, Dallas, TX 75390 USA
[4]Center for Biological Physics, Department of Physics, Arizona State University, Tempe, AZ 85287 USA
[*]klidke@unm.edu

ABSTRACT

We describe a robust, fiducial-free method of drift correction for use in single molecule localization-based super-resolution methods. The method combines periodic 3D registration of the sample using brightfield images with a fast post-processing algorithm that corrects residual registration errors and drift between registration events. The method is robust to low numbers of collected localizations, requires no specialized hardware, and provides stability and drift correction for an indefinite time period.

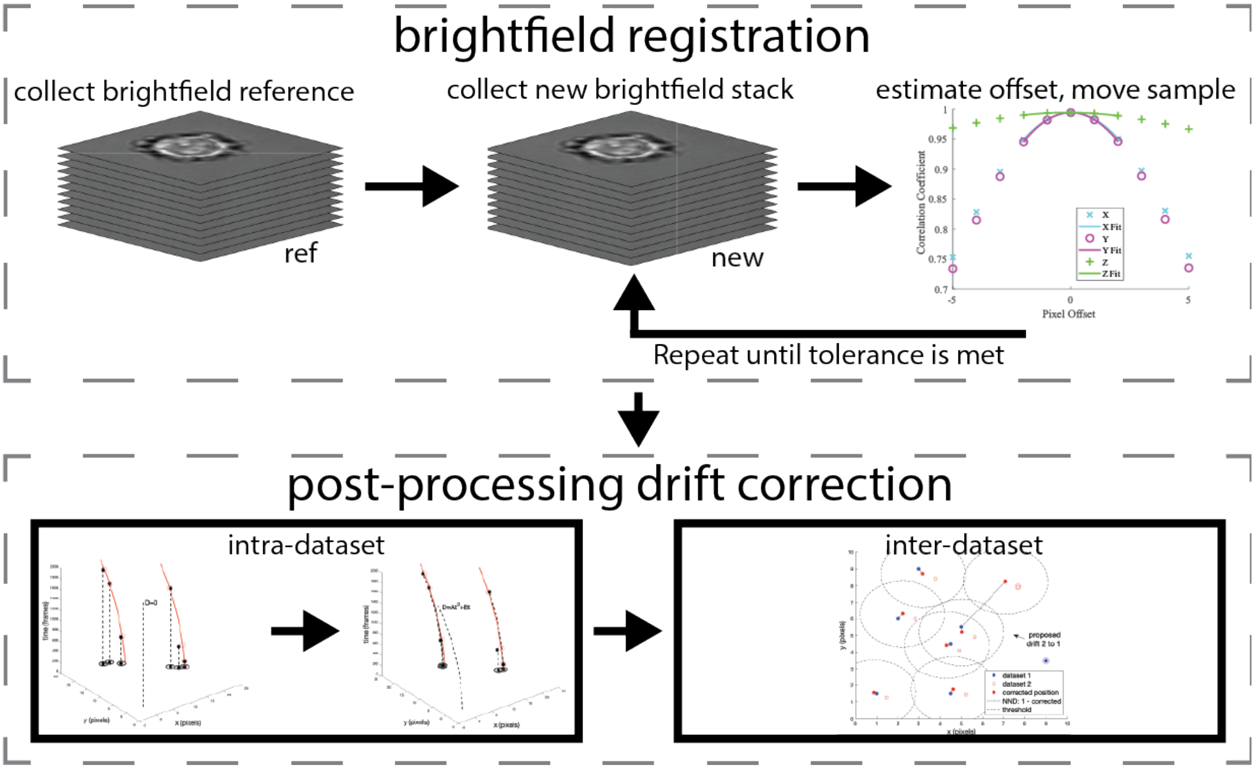

Figure S1. Flow chart of the sequence of steps we recommend to minimize drift. (1) After obtaining a reference brightfield *z*-stack before any data collection, perform brightfield registration at regular intervals (dataset boundaries). This involves collecting a new brightfield *z*-stack to compare to the reference *z*-stack, estimating the offset, then moving the sample, repeatedly performing this sequence until a user tolerance is met. The supplied MATLAB code findStackOffset can be used to estimate the offset. We suggest ∼2,000 frames/dataset for dSTORM data and ∼1,000 frames/dataset for DNA-PAINT data. (2) After data collection, using the datasets established during brightfield registration, perform (a) intra-dataset and then (b) inter-dataset post-processing drift correction, for example, by using the supplied MATLAB code driftCorrectKNN. If a very large dataset size was initially chosen, the MATLAB algorithm under user direction can internally reorganize the datasets into fewer numbers of frames, sometimes improving the results. Please see Fig. 1, and sections **Algorithm** and **Results; Breaking up datasets** for more details.

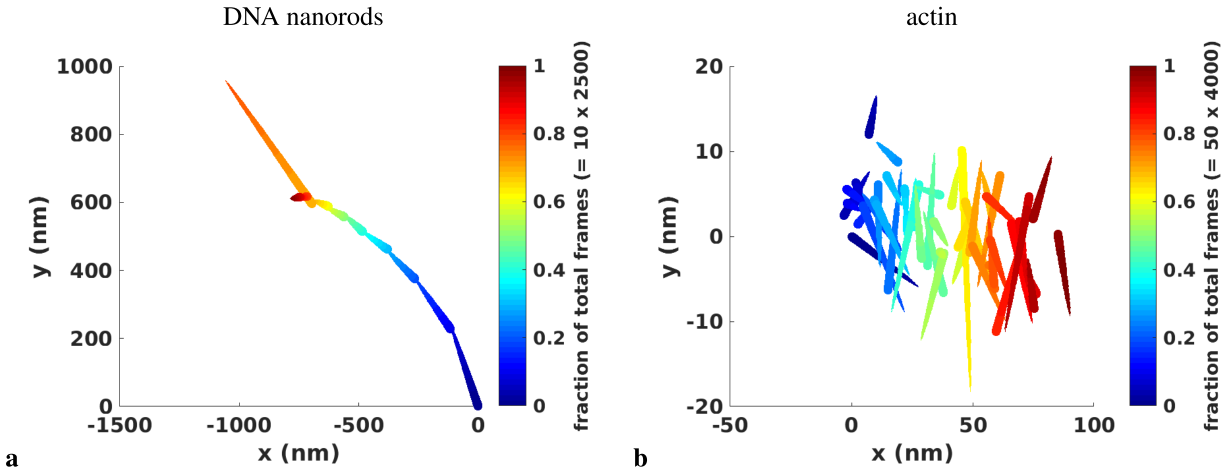

Figure S2. 2D estimated drift plots. Each dataset is represented by a separate line segment. Frames are color coded from blue to red to indicate the passage of time. Within each dataset, passage of time is also indicated by the segment width tapering from large to small (like an arrowhead). (a) 80 nm nanorods with spots 40 nm apart produced by DNA-PAINT. The initial guess used for the inter-dataset optimization procedure was the drift-corrected values from the last frame (2500) of each previous dataset. (b) Actin microfilaments in HeLa cells. The initial guess used for the inter-dataset optimization procedure was zero.

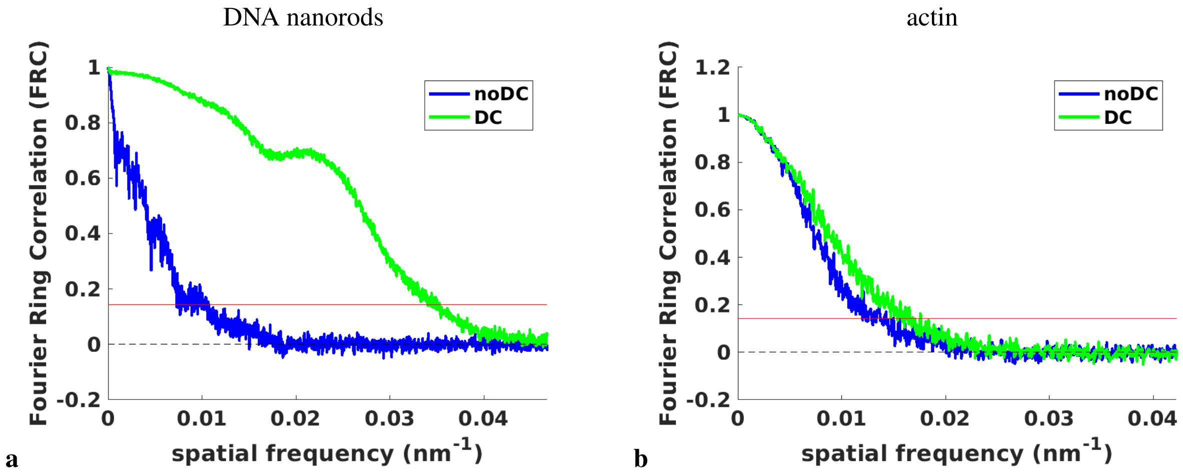

Figure S3. Fourier Ring Correlation plots. (a) 80 nm nanorods with spots 40 nm apart produced by DNA-PAINT. (b) Actin microfilaments in HeLa cells. The blue lines represent raw (uncorrected) data, while the green lines are the results given data drift corrected through the post-processing algorithm. The black horizontal line at $\mathrm{FRC} = \frac{1}{7}$ is the threshold that when crossed by the FRC curve defines the spatial resolution of the image, as the inverse of the spatial frequency at the crossing point. For (a), the estimated resolutions of the uncorrected data compared to the corrected results were 97.2 ± 0.9 nm versus 28.7 ± 0.1 nm, while for (b), these numbers were 72.5 ± 1.4 nm and 60.9 ± 0.6 nm, respectively.

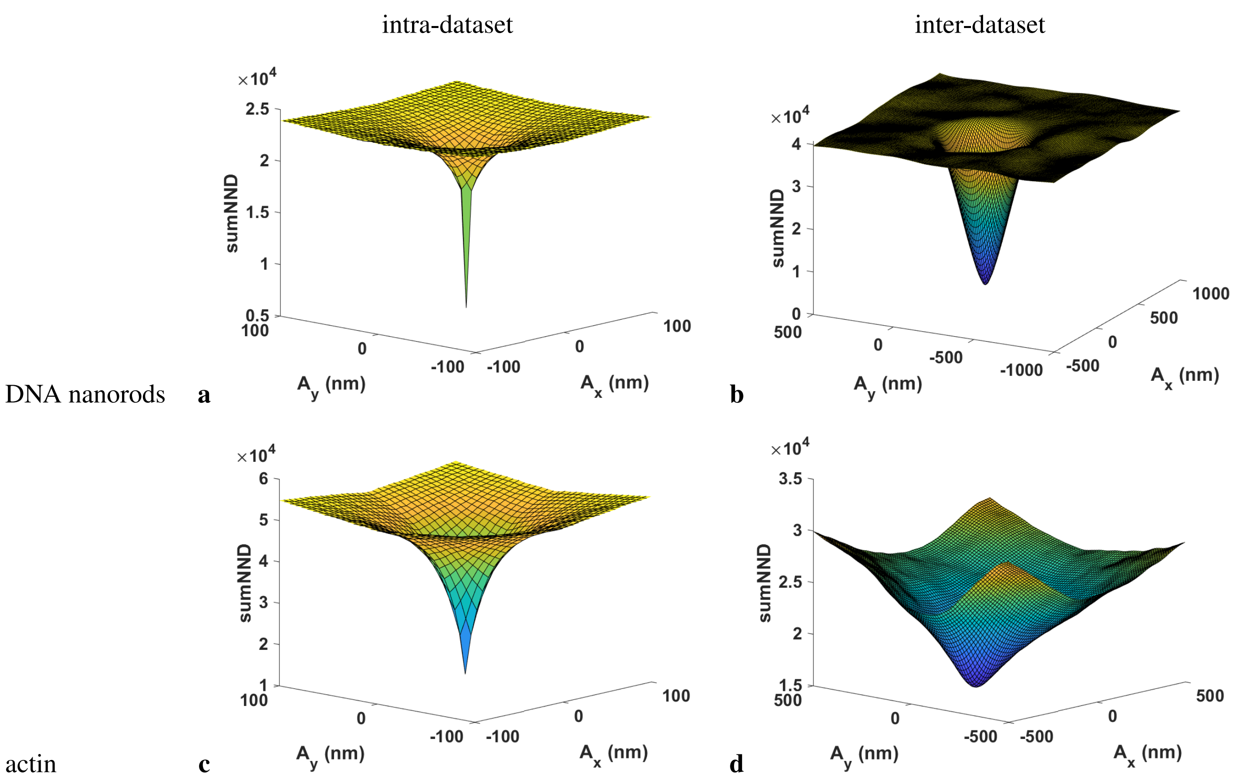

Figure S4. Cost function landscapes for driftCorrectKNN when fitting a linear polynomial with zero constant term to the intra-dataset drift correction and a constant lateral shift to the inter-dataset drift correction. (a,b) Dataset of 80 nm nanorods with spots 40 nm apart produced by DNA-PAINT. (c,d) Dataset of actin microfilaments in HeLa cells. (a,c) Intra-dataset landscape for the first dataset. The (x,y) coordinates refer to the coefficients of the linear (and only) term in the polynomial fit of the intra-dataset drift correction. sumNND (defined by Eq. 1) is the cost. (b,d) Inter-dataset landscape for the first dataset processed (dataset 2 which is shifted relative to dataset 1). The (x,y) coordinates refer to the constant lateral x/y shifts of the inter-dataset drift correction.

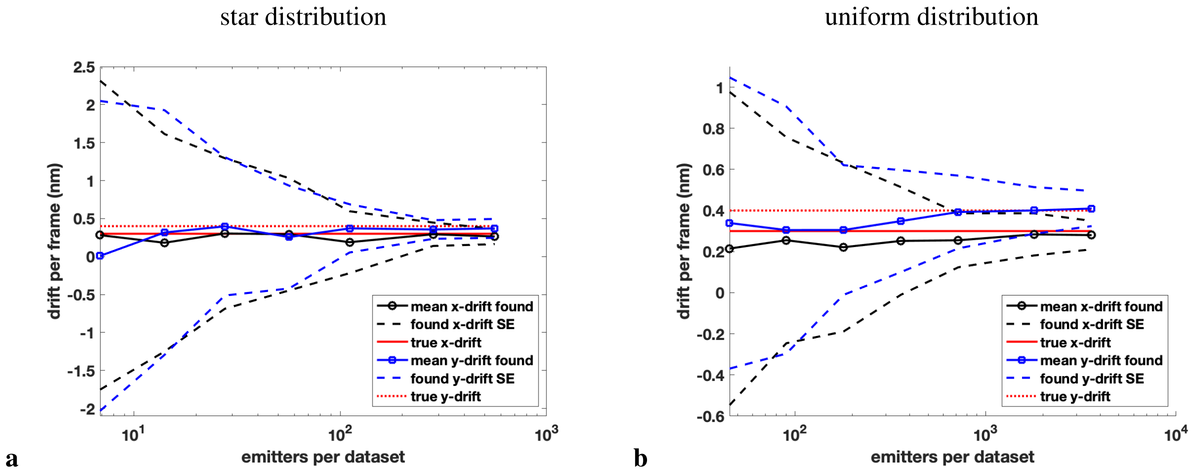

Figure S5. Results of drift correction for true constant shifts applied to noisy random emitters occupying two differently shaped domains as a function of the number of emitters per dataset. (a) 2D star-shaped domain. (b) 2D uniformly distributed domain. 10 datasets of 100 frames each were generated per simulation. A drift of $\Delta x = 0.3$ and $\Delta y = 0.4$ nm/frame was then applied. The solid black and blue lines are simulation means, while the dashed black and blue lines define one standard deviation about the means. The results were averaged over $N = 100$ simulations for each condition.

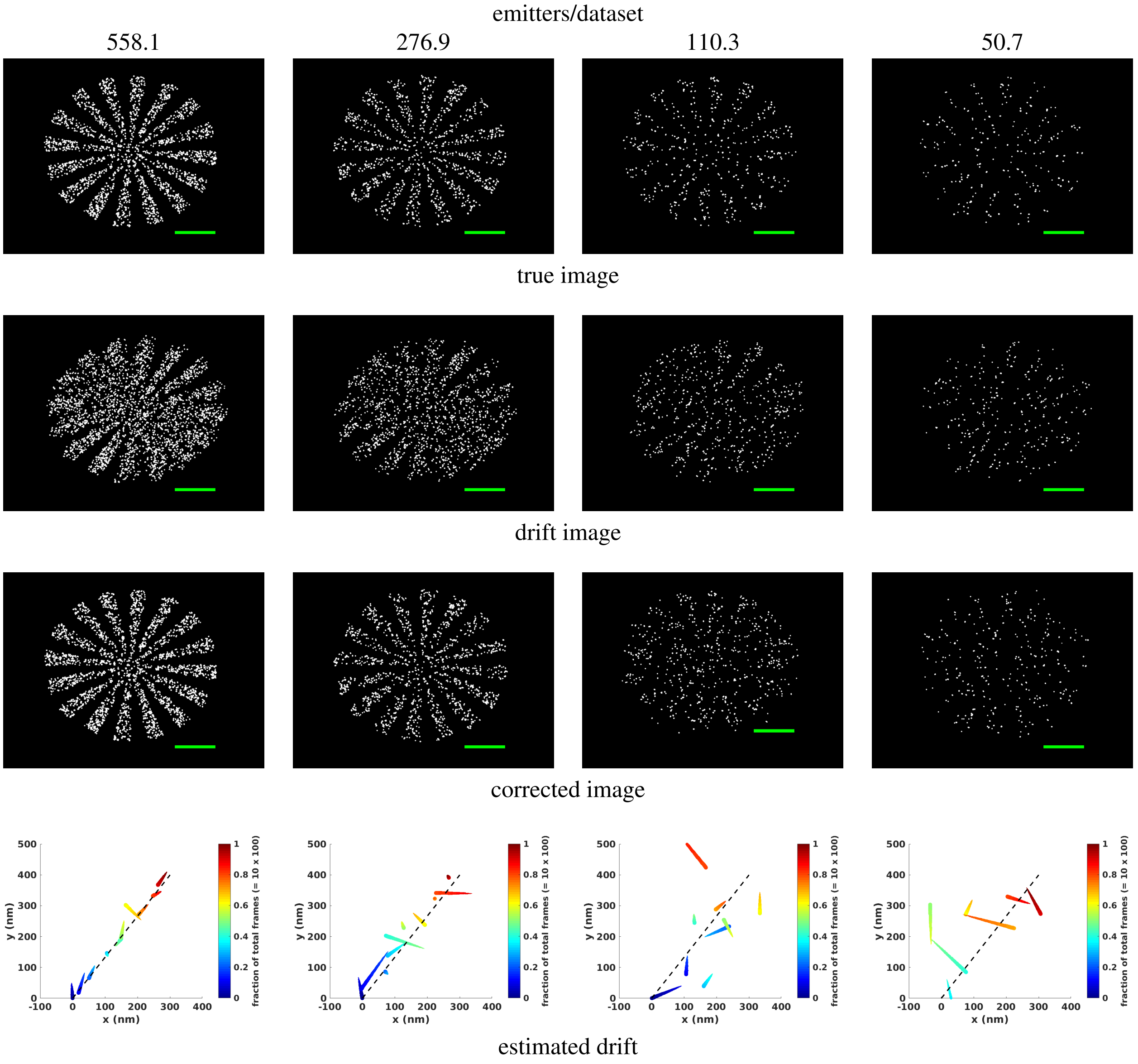

Figure S6. Actions of driftCorrectKNN on a drifted 2D star-shaped domain for various densities of (noisy) emitters per dataset. (1st row) True images of the original random emitters confined to a star-shaped domain. 10 datasets of 100 frames each were generated. (2nd row) Drifted images where a drift of $\Delta x = 0.3$ and $\Delta y = 0.4$ nm/frame was applied. (3rd row) The drift-corrected images produced by driftCorrectKNN using default settings. (4th row) Estimated drift plots. The black dashed lines depict the true drifts. All scale bars are 1 μm.

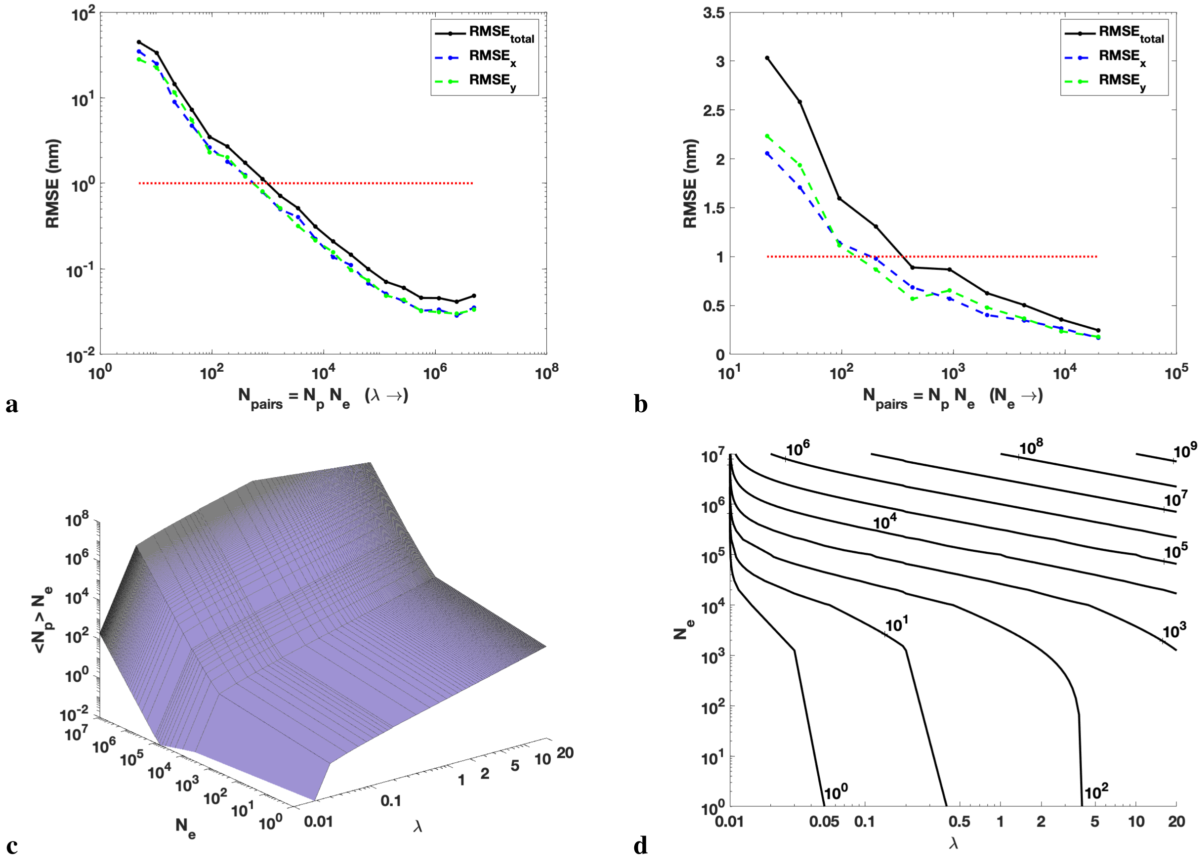

Figure S7. Fluorophore pairings for intra-dataset drift correction. (a,b) RMSE (and x and y components) between the true and the estimated drift curves plotted versus the number of pairs of blinking events, N_pN_e, for noisy 2D uniform randomly distributed emitters with (a) λ increasing from 0.01–10 and fixed $N_e = 10^5$, (b) N_e increasing from 10^3–10^6 and fixed $\lambda = 0.2$. The imposed drift was linear and the intra-dataset fitting was also linear. Results were averaged over $N = 100$ simulations. The red dotted lines correspond to RMSE = 1 nm. (c) Theoretical 3D plot of $< N_p > N_e$ versus the number of expected blinking events per emitter, λ, and the number of emitters, N_e, for a dataset. (d) 2D contour plot of the surface in which lines of constant $< N_p > N_e$ are displayed.

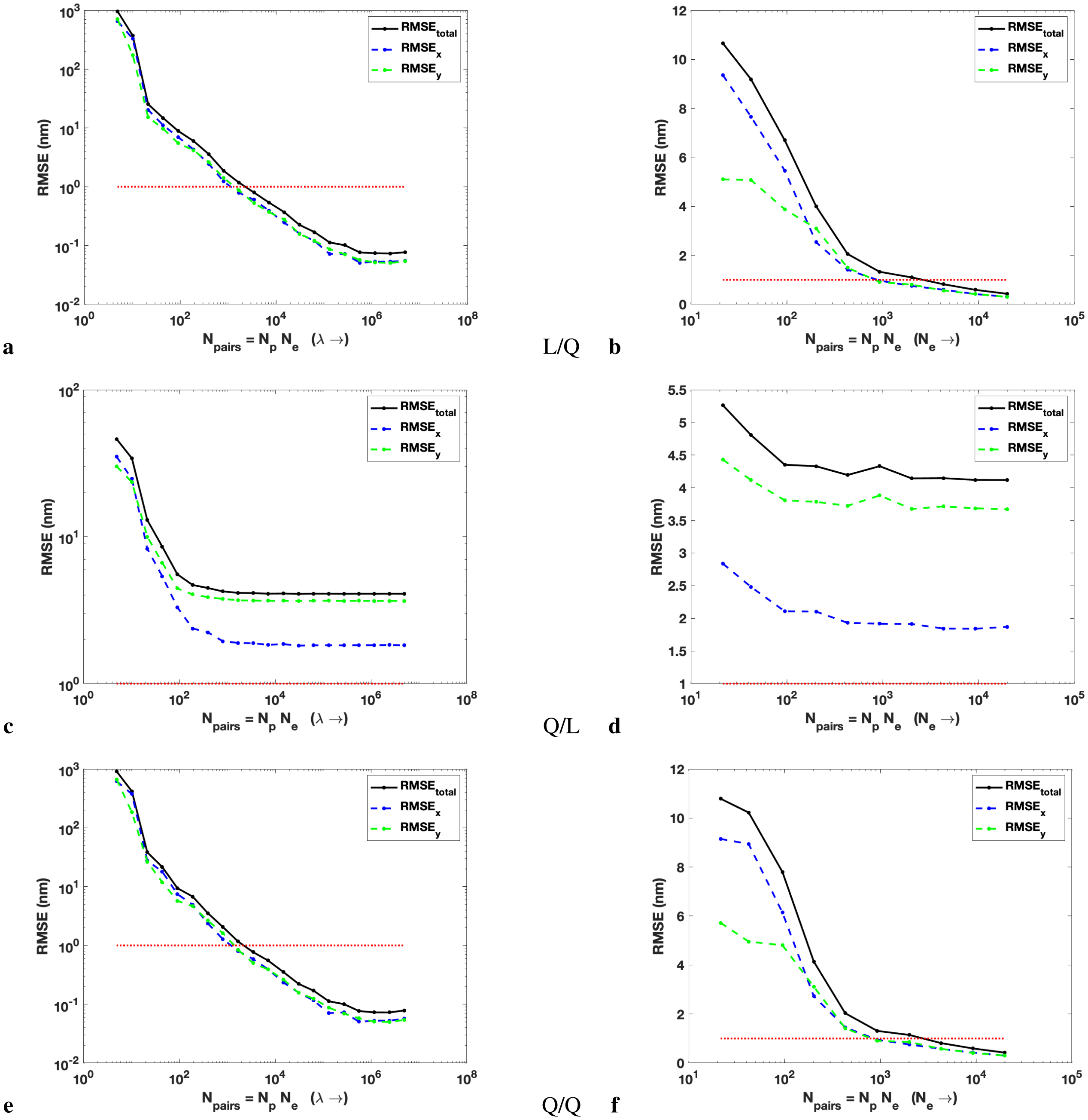

Figure S8. Fluorophore pairings for intra-dataset drift correction. RMSE (and x and y components) between the true and the estimated drift curves plotted versus the number of pairs of blinking events, N_pN_e, for noisy 2D uniform randomly distributed emitters with (a,c,e) λ increasing from 0.01–10 and fixed $N_e = 10^5$, (b,d,f) N_e increasing from 10^3–10^6 and fixed $\lambda = 0.2$. The imposed drift was (a,b) linear / (c,d,e,f) quadratic, and the intra-dataset fitting was (c,d) linear / (a,b,e,f) quadratic. This is also indicated by L/Q (linear imposed drift/quadratic intra-dataset fitting), etc. above. Results were averaged over $N = 100$ simulations. The red dotted lines correspond to RMSE = 1 nm.

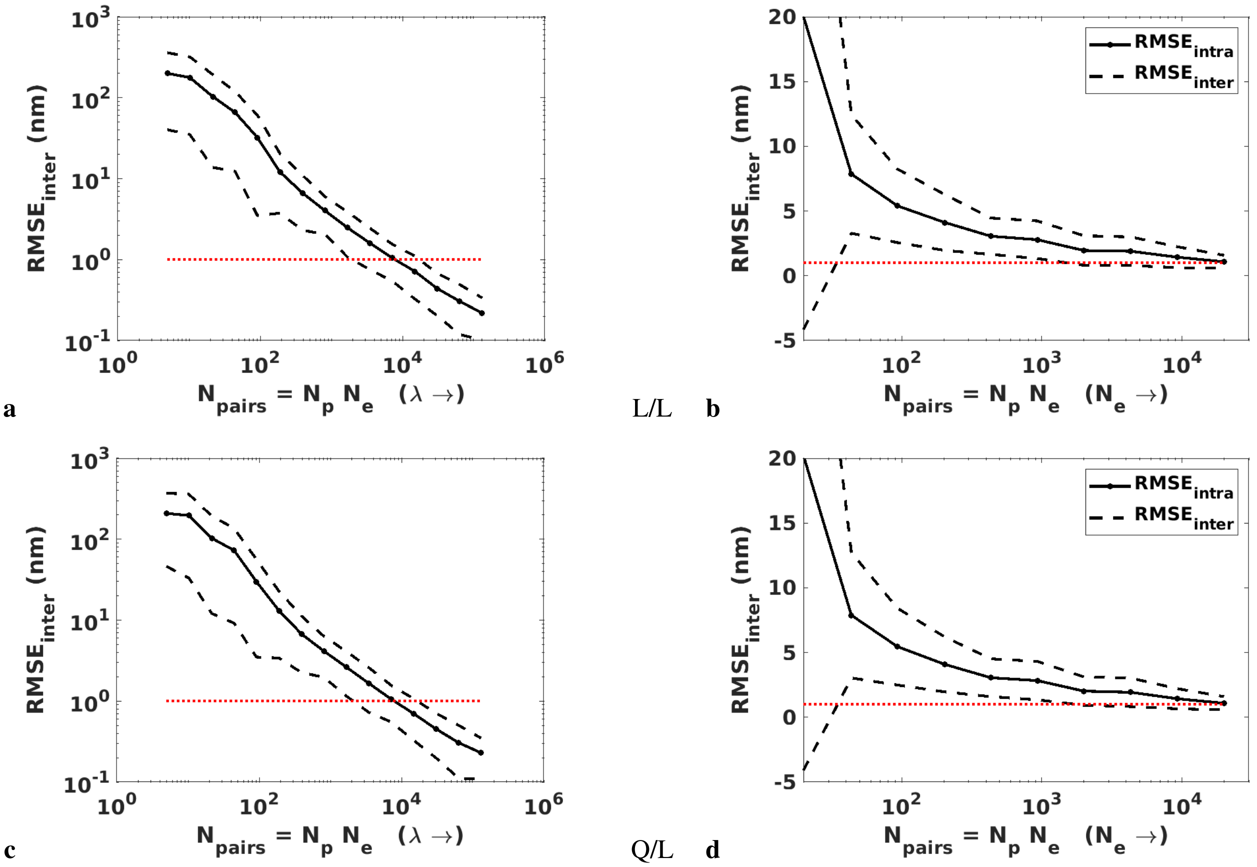

Figure S9. Fluorophore pairings for inter-dataset drift correction. Here, a 2,000 frame data collection was broken up into two datasets. RMSE between the true and the estimated drift curves plotted versus the number of pairs of blinking events, N_pN_e, for noisy 2D uniform randomly distributed emitters with (a,c) λ increasing from 0.01–1.6 and fixed $N_e = 10^5$, (b,d) N_e increasing from 10^3–10^6 and fixed $\lambda = 0.2$. The imposed drift was (a,b) linear / (c,d) quadratic, and the intra-dataset fitting was linear. This is also indicated by Q/L (quadratic imposed drift/linear intra-dataset fitting), etc. above. Results were averaged over $N = 100$ simulations. The red dotted lines correspond to RMSE = 1 nm. The dashed lines indicate one standard deviation from the mean (solid lines).

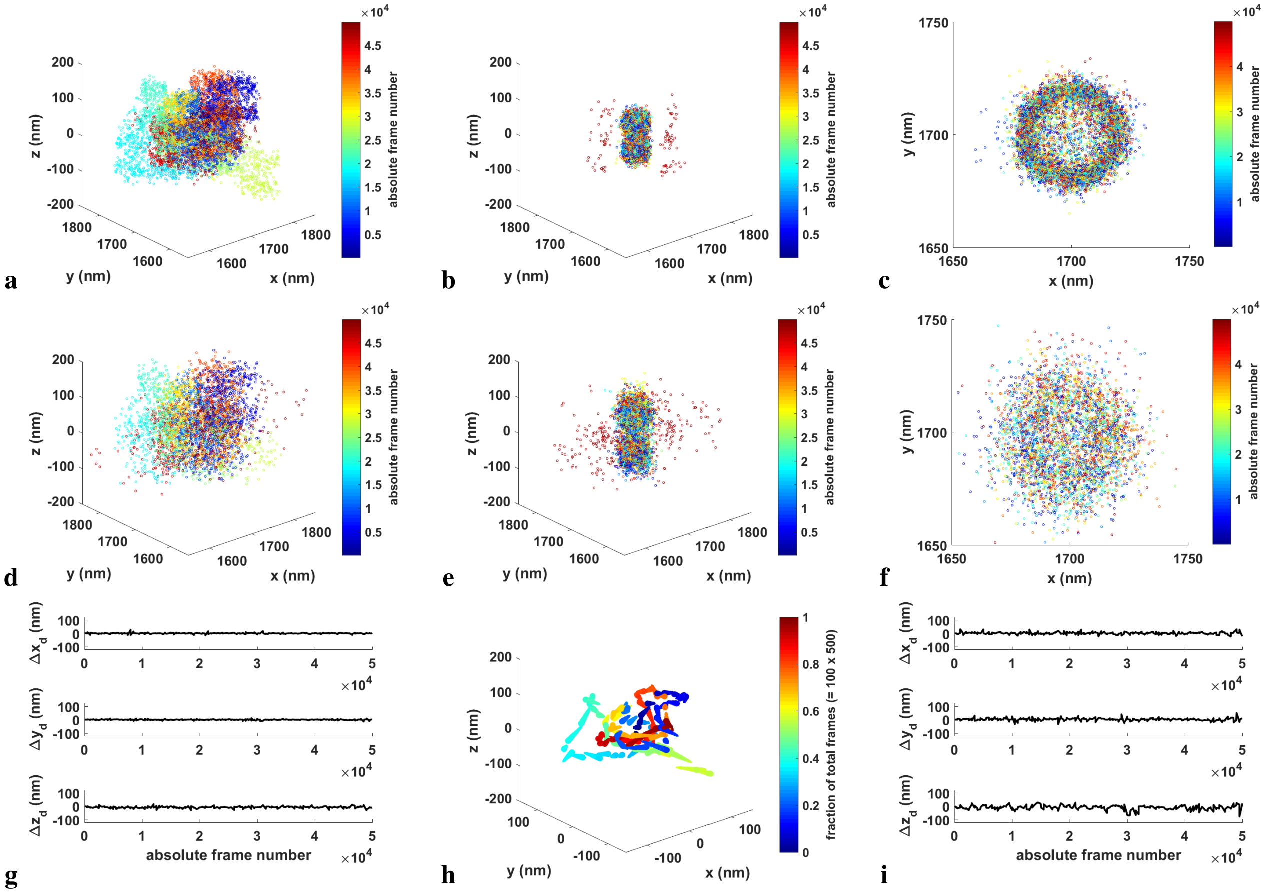

Figure S10. 3D drift correction applied to simulated 40 nm diameter rings separated by 80 nm (a,b,c,g,h) or 120 nm (d,e,f,i). A simulated/experimental PSF for the 80 nm/120 nm separated rings was used, along with simulated drift curves in both cases. 50,000 frames were generated, divided into 100 datasets of 500 frames each. To make the simulation more realistic, the simulated localizations were fit and thresholded after being drifted. (a,d) Drifted image. The emitters are color-coded by frame number. (b,e) The drift-corrected image produced by driftCorrectKNN using default settings. (c,f) x, y perspective of the drift-corrected image. (g,i) The difference between the estimated and simulated x, y, z-drifts as a function of the absolute frame number for the (g/i) 80 nm/120 nm separated rings. (h) Corresponding drift correction plot for the 80 nm separation example.

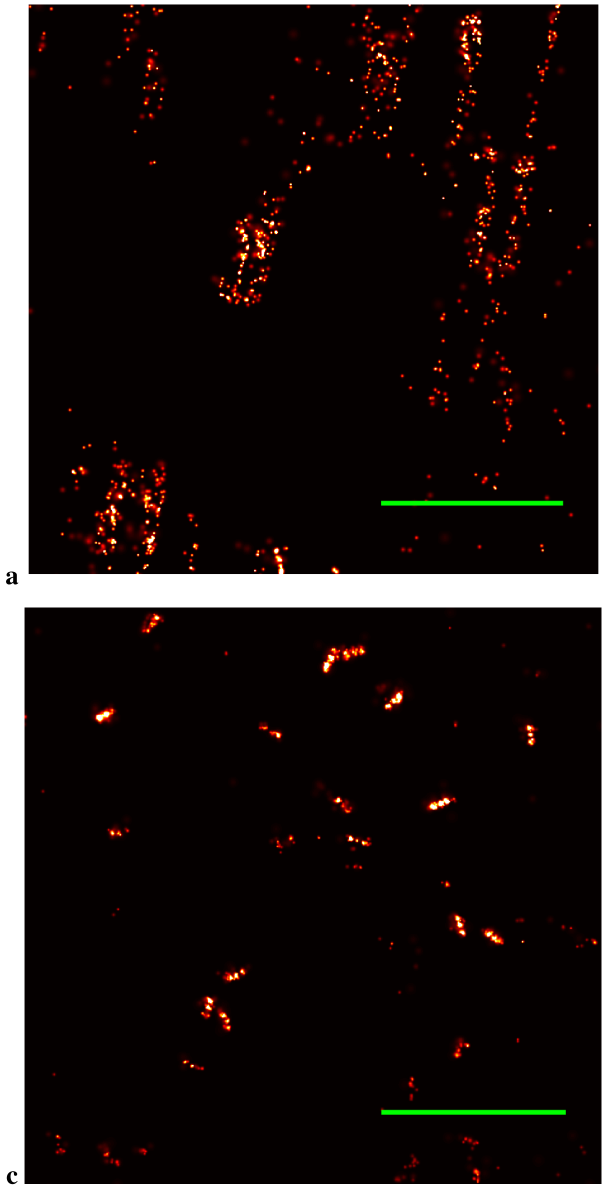

Figure S11. Example of internally reorganizing the datasets from an experiment examining ATTO655 20 nm nanorulers. The data was initially collected in a single dataset consisting of 15,000 frames. (a) Raw super-resolution image. (b) Post-processing drift correction was applied to one 15,000-frame dataset. (c) Post-processing drift correction was applied to 15 1,000-frame datasets. (d) Post-processing drift correction was applied to 150 100-frame datasets. All scale bars measure 1 μm.

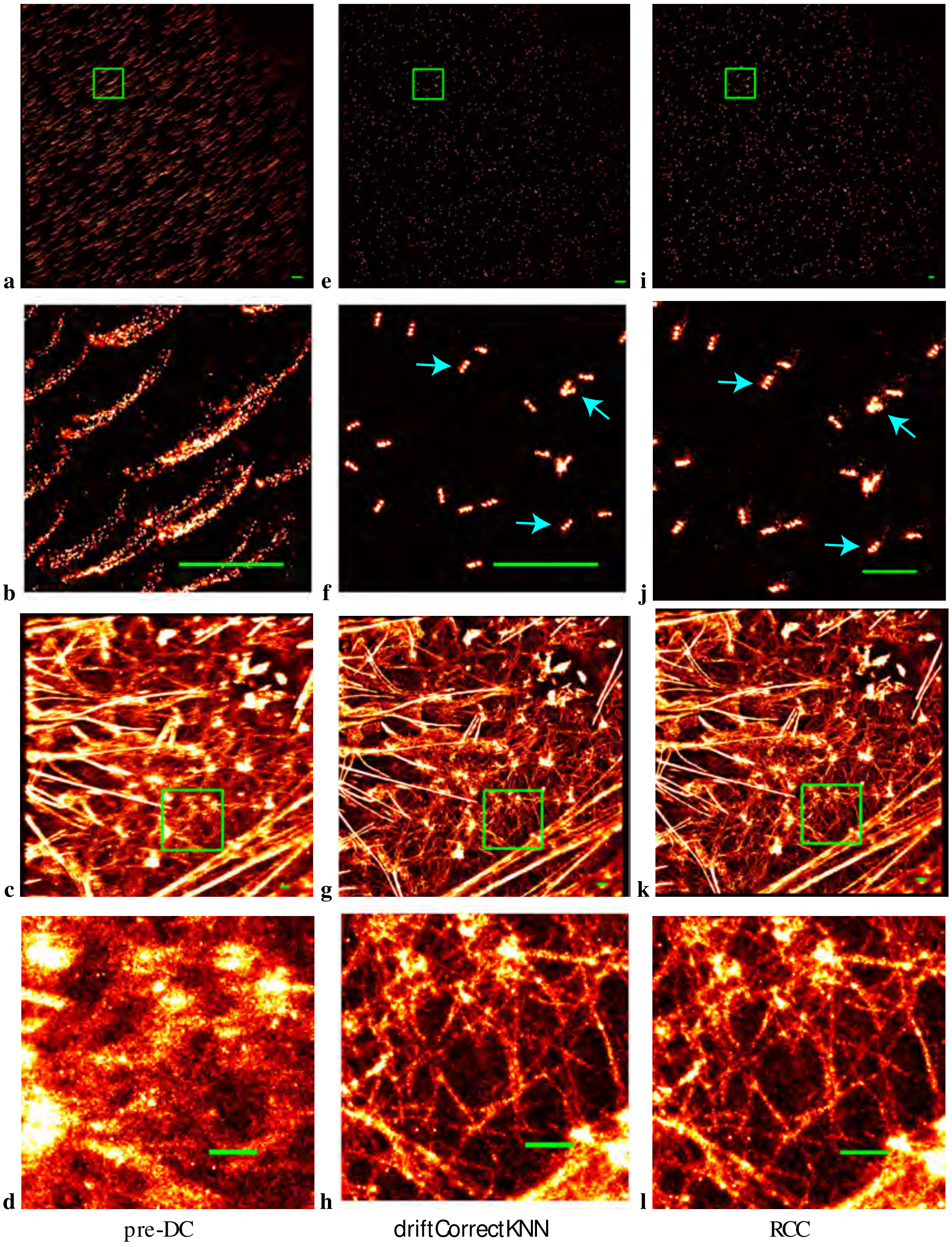

Figure S12. 2D RCC drift correction for examples of DNA-PAINT and reversibly binding Lifeact localizations, the latter which have been adjusted to minimize brightfield registration effects (see text). These are compared with the results from Fig. 3. (a,e,i) 80 nm nanorods with spots 40 nm apart produced by DNA-PAINT. (b,f,j) Zoomed in view of the selected region in the previous images. (c,g,k) Actin microfilaments in HeLa cells. (d,h,l) Zoomed in view of the selected region in the previous images. (a–d) Pre-drift corrected (pre-DC) image. (e–h) Drift corrected image using driftCorrectKNN. (i–l) Drift corrected image using RCC. The blue arrows point to areas where differences in resolution are noticeable between driftCorrectKNN and RCC. All scale bars measure 1 μm.

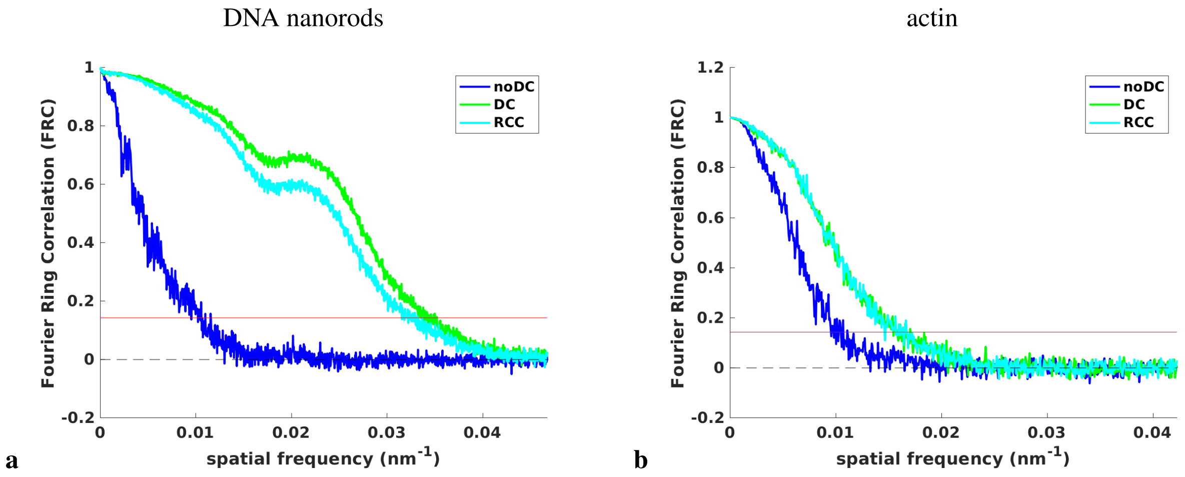

Figure S13. Fourier Ring Correlation plots. (a) 80 nm nanorods with spots 40 nm apart produced by DNA-PAINT. (b) Actin microfilaments in HeLa cells in which the image has been adjusted to minimize the effects of brightfield registration (see text). The blue lines represent raw (uncorrected) data, while the green lines are the results given data drift corrected through the post-processing algorithm. Similarly, the cyan lines are the results after processing through RCC. The black horizontal line at FRC $= \frac{1}{7}$ is the threshold that when crossed by the FRC curve defines the spatial resolution of the image, as the inverse of the spatial frequency at the crossing point. For (a), the estimated resolutions of the uncorrected data compared to the corrected results and data processed through RCC were 95.5 ± 0.6 nm versus 29.1 ± 0.1 nm and 30.7 ± 0.1 nm, while for (b), these numbers were 97.7 ± 1.3 nm, 62.6 ± 0.7 nm and 61.0 ± 0.7 nm, respectively. Note also in (b) that the DC and RCC curves overlay each other. Compare with Supplementary Fig. S3.

frames/ dataset	x,y-drift/frame mean dCK (nm)		bin size (nm)	Δ_{rmax} (pixel)	x,y-drift/frame mean RCC (nm)	
true	0.400	0.700			0.400	0.700
50	0.364	0.876	16	0.2	0.431	0.363
			32	0.2	0.089	0.257
			48	0.2	0.447	0.663
			64	0.2	—	—
100	0.434	0.771	4	0.2	0.059	0.073
			8	0.2	-0.032	0.187
			16	0.2	0.974	0.320
			32	0.2	0.266	0.515
			48	0.2	0.272	0.510
			64	0.2	0.263	0.548
			64	1	0.267	0.525
			64	2	0.267	0.522
			64	5	0.267	0.522
			64	10	0.267	0.522
			64	20	0.267	0.522
125	0.451	0.713	16	0.2	0.259	0.503
			32	0.2	0.271	0.038
			48	0.2	0.286	0.474
			64	0.2	0.251	0.501
200	0.451	0.687	16	0.2	0.239	0.425
			32	0.2	0.240	0.414
			48	0.2	0.241	0.413
			64	0.2	0.236	0.411
250	0.410	0.675	16	0.2	0.212	0.381
			32	0.2	0.215	0.381
			48	0.2	0.217	0.379
			64	0.2	0.212	0.377
500	0.112	0.217	16	0.2	0.114	0.198
			32	0.2	0.114	0.196
			48	0.2	0.115	0.196
			64	0.2	0.114	0.196
1000	0.397	0.699			—	—

Table S1. Results of driftCorrectKNN (dCK) and RCC applied to a simulated 64×64 pixel star-shaped data collection containing 5,592 localizations and consisting of 1000 frames. The data collection was broken up into different numbers of frames per dataset (leftmost column), and different spatial bin sizes and Δ_{rmax} for RCC (third and fourth columns), with results given in subsequent columns for mean x- and y-drift per frame (nm) by dCK and RCC. Dashes (—) indicate RCC was unable to perform the specified correction. Here, 1 pixel = 100 nm. The true drift per frame in the simulations is given in the top row.

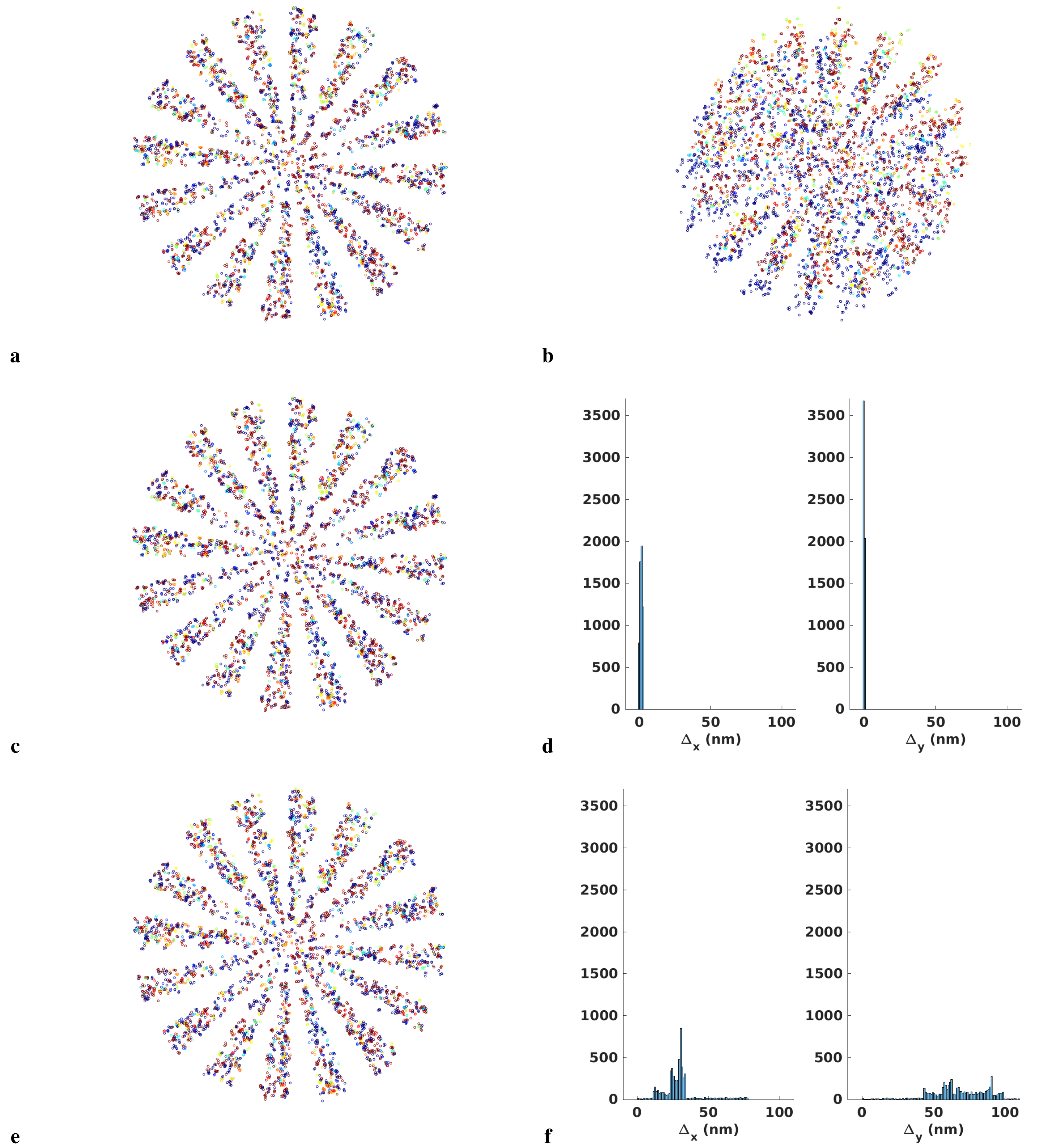

Figure S14. Best results of driftCorrectKNN (dCK) and RCC applied to a simulated 64×64 pixel star-shaped data collection containing 5,714 localizations and consisting of 1,000 frames. For this example, there are ~108.5 emitters/μm^2 and $\lambda = 1.27$ blinking events/emitter. (a) True image. (b) Drift image. (c) dCK corrected drift image using 1,000 frame datasets. (d) Histograms of computed dCK localization positions minus their true values for x and y (Δ_x, Δ_y). (e) RCC corrected drift image using 125 frame datasets and a spatial bin size of 48 nm. (f) Histograms of computed RCC localization positions minus their true values for x and y (Δ_x, Δ_y). Dots are color coded from blue to red to indicate increasing localization appearance time counted by their absolute frame number.

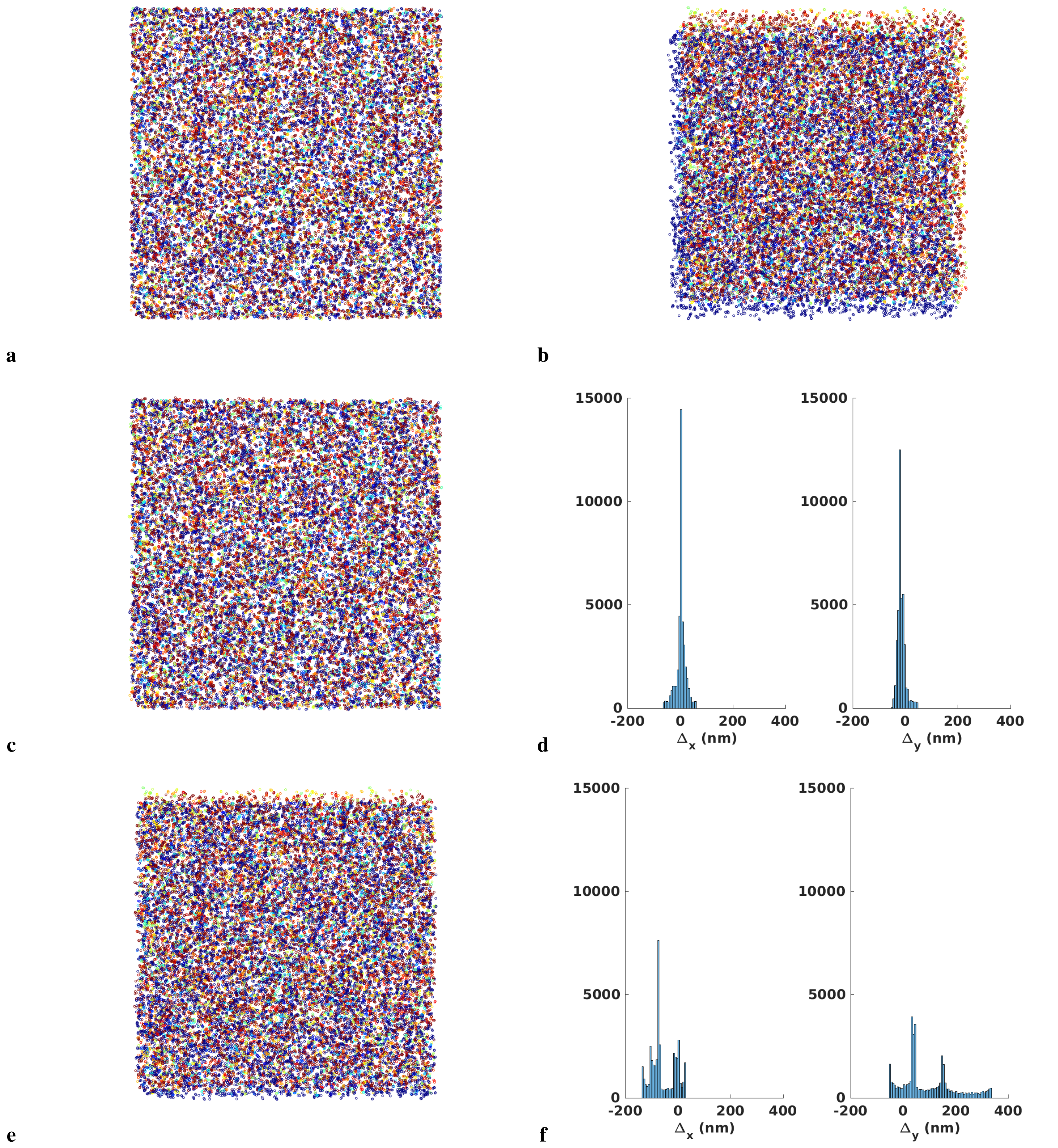

Figure S15. Best results of driftCorrectKNN (dCK) and RCC applied to a simulated 64×64 pixel square-shaped data collection containing 39,829 localizations and consisting of 1,000 frames. For this example, there are ∼382.9 emitters/μm^2 and $\lambda = 1.28$ blinking events/emitter. (a) True image. (b) Drift image. (c) dCK corrected drift image using 125 frame datasets. (d) Histograms of computed dCK localization positions minus their true values for x and y (Δ_x, Δ_y). (e) RCC corrected drift image using 125 frame datasets and a spatial bin size of 48 nm. (f) Histograms of computed RCC localization positions minus their true values for x and y (Δ_x, Δ_y). Dots are color coded from blue to red to indicate increasing localization appearance time counted by their absolute frame number.

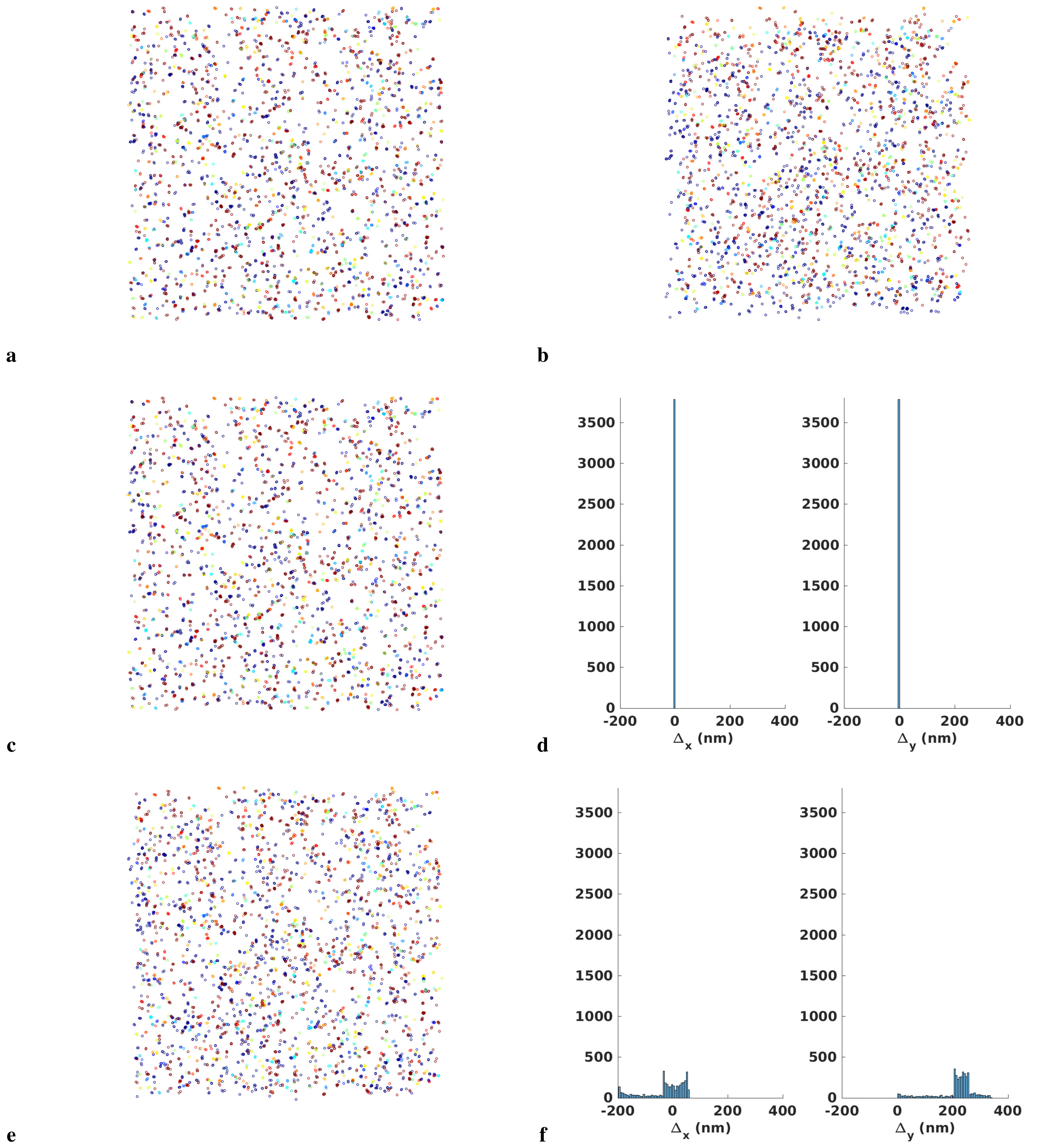

Figure S16. Best results of driftCorrectKNN (dCK) and RCC applied to a simulated 64×64 pixel square-shaped data collection containing 3,784 localizations and consisting of 1,000 frames. For this example, there are ∼36.5 emitters/μm^2 and $\lambda = 1.29$ blinking events/emitter. (a) True image. (b) Drift image. (c) dCK corrected drift image using 1,000 frame datasets. (d) Histograms of computed dCK localization positions minus their true values for x and y (Δ_x, Δ_y). (e) RCC corrected drift image using 200 frame datasets and a spatial bin size of 16 nm. (f) Histograms of computed RCC localization positions minus their true values for x and y (Δ_x, Δ_y). Dots are color coded from blue to red to indicate increasing localization appearance time counted by their absolute frame number.

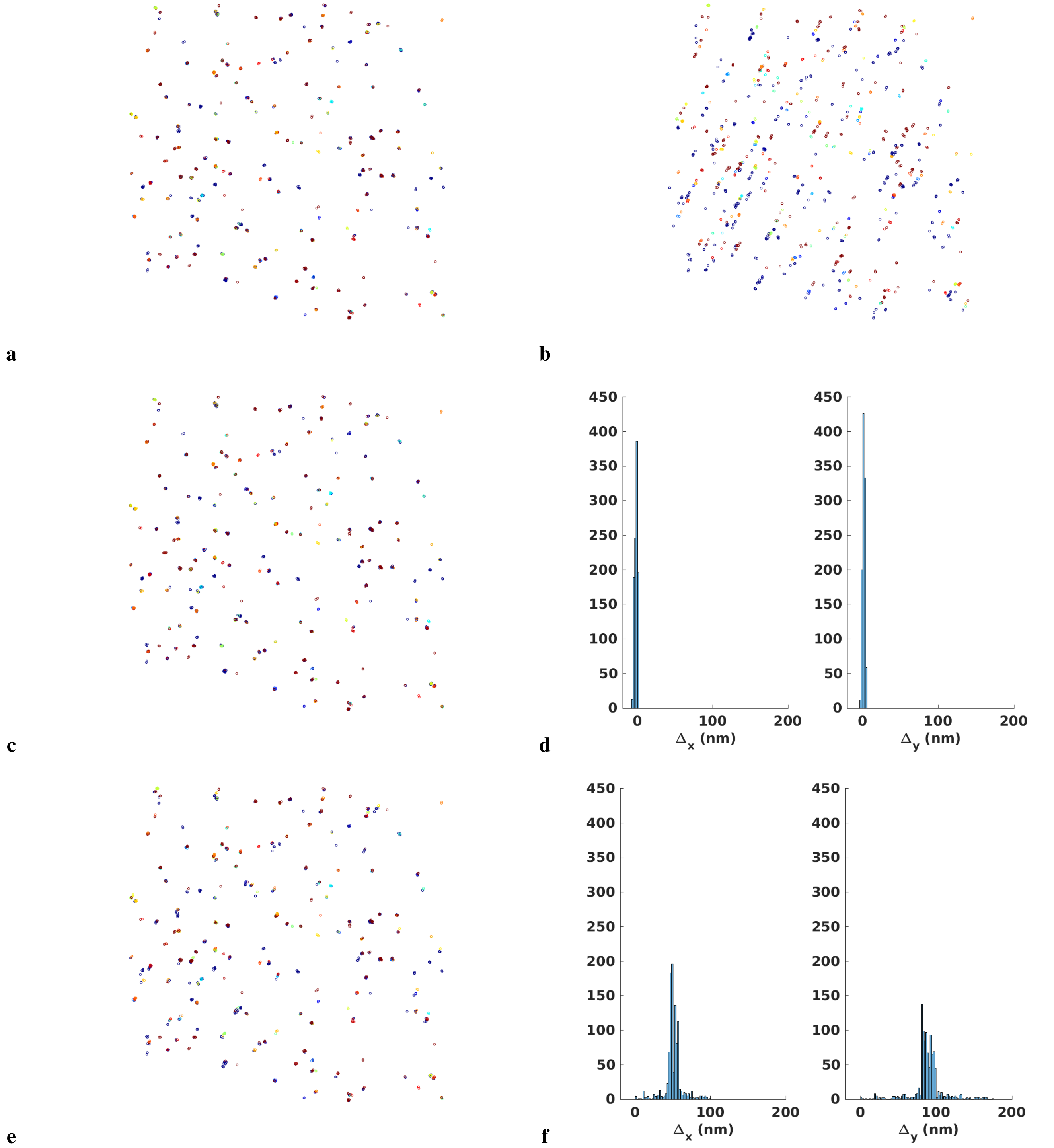

Figure S17. Best results of driftCorrectKNN (dCK) and RCC applied to a simulated 64×64 pixel square-shaped data collection containing 1,030 localizations and consisting of 1,000 frames. For this example, there are ∼4.5 emitters/μm^2 and $\lambda = 2.78$ blinking events/emitter. (a) True image. (b) Drift image. (c) dCK corrected drift image using 500 frame datasets. (d) Histograms of computed dCK localization positions minus their true values for x and y (Δ_x, Δ_y). (e) RCC corrected drift image using 200 frame datasets and a spatial bin size of 32 nm. (f) Histograms of computed RCC localization positions minus their true values for x and y (Δ_x, Δ_y). Dots are color coded from blue to red to indicate increasing localization appearance time counted by their absolute frame number.

Methods

Frame connection

A blinking event often produces localizations across a sequence of frames. These localizations can be recognized as a single fluorophore and connected together to produce better precision. Frame connection takes the time-ordered localizations computed from a super-resolution dataset and attempts to combine them via a single emitter model in which the maximum distance and maximum frame gap between two localizations are specified (defaults are 1 pixel and 4 frames). The level of significance (LoS, default is 0.01) represents the minimum probability for which the null hypothesis that the two localizations come from a single emitter is not rejected. If the value computed for the probability is greater than the LoS and the other conditions hold, then the two localizations are combined. This process examines all localizations that satisfy the distance and frame gap constraints.

If two localizations have positions and localization errors (x_1, σ_1) and (x_2, σ_2), then the combined position and error is given by

$$x' = \frac{\frac{x_1}{\sigma_1^2} + \frac{x_2}{\sigma_2^2}}{\frac{1}{\sigma_1^2} + \frac{1}{\sigma_2^2}} \quad \text{and} \quad \sigma' = \sqrt{\frac{1}{\frac{1}{\sigma_1^2} + \frac{1}{\sigma_2^2}}}, \quad \text{therefore,} \quad \log R = -\frac{1}{2}\left[\left(\frac{x_1 - x'}{\sigma_1}\right)^2 + \left(\frac{x_2 - x'}{\sigma_2}\right)^2\right]$$

is the log-likelihood ratio (minus a constant term) that the two localizations represent a single emitter. The likelihood ratio is given by $R = \frac{L(D|\theta')}{L(D|D)}$, where the numerator is the likelihood L of the data D given the parameters θ' described above for the single emitter model, and the denominator is the likelihood of D given D, or one. L comes from the product of two Gaussians:

$$L = \left(\frac{1}{\sqrt{2\pi\sigma_1^2}} e^{-\frac{(x_1 - x')^2}{2\sigma_1^2}}\right)\left(\frac{1}{\sqrt{2\pi\sigma_2^2}} e^{-\frac{(x_2 - x')^2}{2\sigma_2^2}}\right) = \frac{1}{2\pi\sigma_1\sigma_2} e^{-\frac{1}{2}\left[\left(\frac{x_1 - x'}{\sigma_1}\right)^2 + \left(\frac{x_2 - x'}{\sigma_2}\right)^2\right]}.$$

The corresponding p-value (which is compared to the LoS) is

$$p\text{-value} = 1 - \chi^2_{\mathrm{CDF}}(N_{\mathrm{DoF}}, -2\log R) = 1 - \frac{1}{\Gamma\left(\frac{N_{\mathrm{DoF}}}{2}\right)} \gamma\left(\frac{N_{\mathrm{DoF}}}{2}, \frac{-2\log R}{2}\right),$$

where N_{DoF} is the number of degrees of freedom and hence the spatial dimension, N_{dim}, in the situation when two localizations are combined into one ($N_{\mathrm{DoF}} = N^{\mathrm{data}}_{\mathrm{DoF}} - N^{\mathrm{model}}_{\mathrm{DoF}}$). Γ and γ are the gamma function and the lower incomplete gamma function, respectively. For $N_{\mathrm{DoF}} = 1, 2, 3$,

$$\chi^2_{\mathrm{CDF}}(N_{\mathrm{DoF}}, x) = \begin{cases} \mathrm{erf}\sqrt{\frac{x}{2}}, & N_{\mathrm{DoF}} = 1 \\ 1 - e^{-\frac{x}{2}}, & N_{\mathrm{DoF}} = 2 \\ \mathrm{erf}\sqrt{\frac{x}{2}} - \sqrt{\frac{2}{\pi}} e^{-x/2}\sqrt{x}, & N_{\mathrm{DoF}} = 3 \end{cases}$$

Note that in 2D ($N_{\mathrm{DoF}} = 2$), the p-value is exactly R.

Post-processing drift correction algorithm

MATLAB pseudocode drift correction algorithm operating on (x, y) or (x, y, z) localizations. The drift corrected coordinates, X, and the matrix of drift corrections indexed by dataset number and frame number, ΔX, are produced. P_{degree} is the degree of the polynomial fitting the intra-dataset drift correction. Note that the constant term is assumed to be zero. N_{frames} is the number of frames per dataset. ΔX_i is the drift correction for each frame of the ith dataset. $p_{\bullet,j} T^j$ is the product of the jth column of p with the vector T, in which each component is raised to the jth power. X^c are drift corrected coordinates.

Establish problem dimensionality: $N_{\text{dims}} = 2$ or 3
Initialize optimization parameters
Default $l_{\text{intra}} = 1$ pixel and $l_{\text{inter}} = 2$ pixels if not set above
Optionally reorganize dataset scheme
Let $\Delta X = 0$ be an $N_{\text{datasets}} \times N_{\text{frames}}$ matrix
If 3D, convert z units into pixels
Intra-Dataset Drift Correction:
for $i = 1$ **to** N_{datasets}
    Select dataset i coordinate localizations, X_i, and frame numbers, F_i
    $p_0 = 0$ where p_0 is an $N_{\text{dims}} \times P_{\text{degree}}$ matrix
    p = fminsearch(@minD_intra, p_0, X_i, F_i, l_{intra})
    $[\sim, X_i]$ = minD_intra(p, X_i, F_i, l_{intra})
    $T = 1 : N_{\text{frames}}$
    $\Delta X_i = \sum_{j=1}^{P_{\text{degree}}} p_{\bullet,j} T^j$
Inter-Dataset Drift Correction:
for $i = 2$ **to** N_{datasets}
    Either $p_0 = 0$ when employing brightfield registration or
        $p_0 = \Delta X_{i-1,N_{\text{frames}}}$ otherwise
    p = fminsearch(@minD_inter, p_0, X_i, l_{inter})
    $[\sim, X_i]$ = minD_inter(p, X_i, l_{inter})
    $\Delta X_i = \Delta X_{i-1} + p_{\bullet,0}$
$\Delta X = -\Delta X$

$[\text{sumNND}, X^c]$ = minD_intra(p, X, F, l_{intra}):
  $X^c = X + \sum_{j=1}^{P_{\text{degree}}} p_{\bullet,j} F^j$
  D = nearest neighbor distances of all localizations X^c in the current dataset
  sumNND = $\sum_{D \le l_{\text{intra}}} D + \sum_{D > l_{\text{intra}}} l_{\text{intra}} = \sum \min(D, l_{\text{intra}})$

$[\text{sumNND}, X^c]$ = minD_inter(p, X, l_{inter}):
  $X^c = X + p$
  D = nearest neighbor distances of all localizations in dataset 1 versus X^c
  sumNND = $\sum_{D \le l_{\text{inter}}} D + \sum_{D > l_{\text{inter}}} l_{\text{inter}} = \sum \min(D, l_{\text{inter}})$

RMSE analysis

The RMSE analysis, depicted in Supplementary Fig. S7(a,b), Supplementary Fig. S8 and Supplementary Fig. S9, plots the root-mean-square error (RMSE) between the true drift curve, $(X_i, Y_i)_{i=1}^n$, and the estimated drift curve, $(x_i, y_i)_{i=1}^n$, of simulated uniform randomly distributed single datasets of 2D emitters, versus the number of blinking event pairs in the datasets. The RMSE was computed by

$$\text{RMSE} = \sqrt{\frac{\sum_{i=1}^n (X_i - x_i)^2 + (Y_i - y_i)^2}{n}},$$

where a constant drift per frame was applied to the true locations to produce the drifted positions, which were then run through the intra-dataset portion of the post-processing drift correction algorithm. This definition of the RMSE considers the difference between the true drift curves at each time frame (as determined by the simulation) and the found (as determined by the intra-dataset portion of the post-processing drift correction algorithm).
