# Supplementary figures and images for: Robust, fiducial-free drift correction for super-resolution imaging
Source: Sci Rep. 2021 Dec 8;11:23672. doi: 10.1038/s41598-021-02850-7 (PMC8655078; doi:10.1038/s41598-021-02850-7)

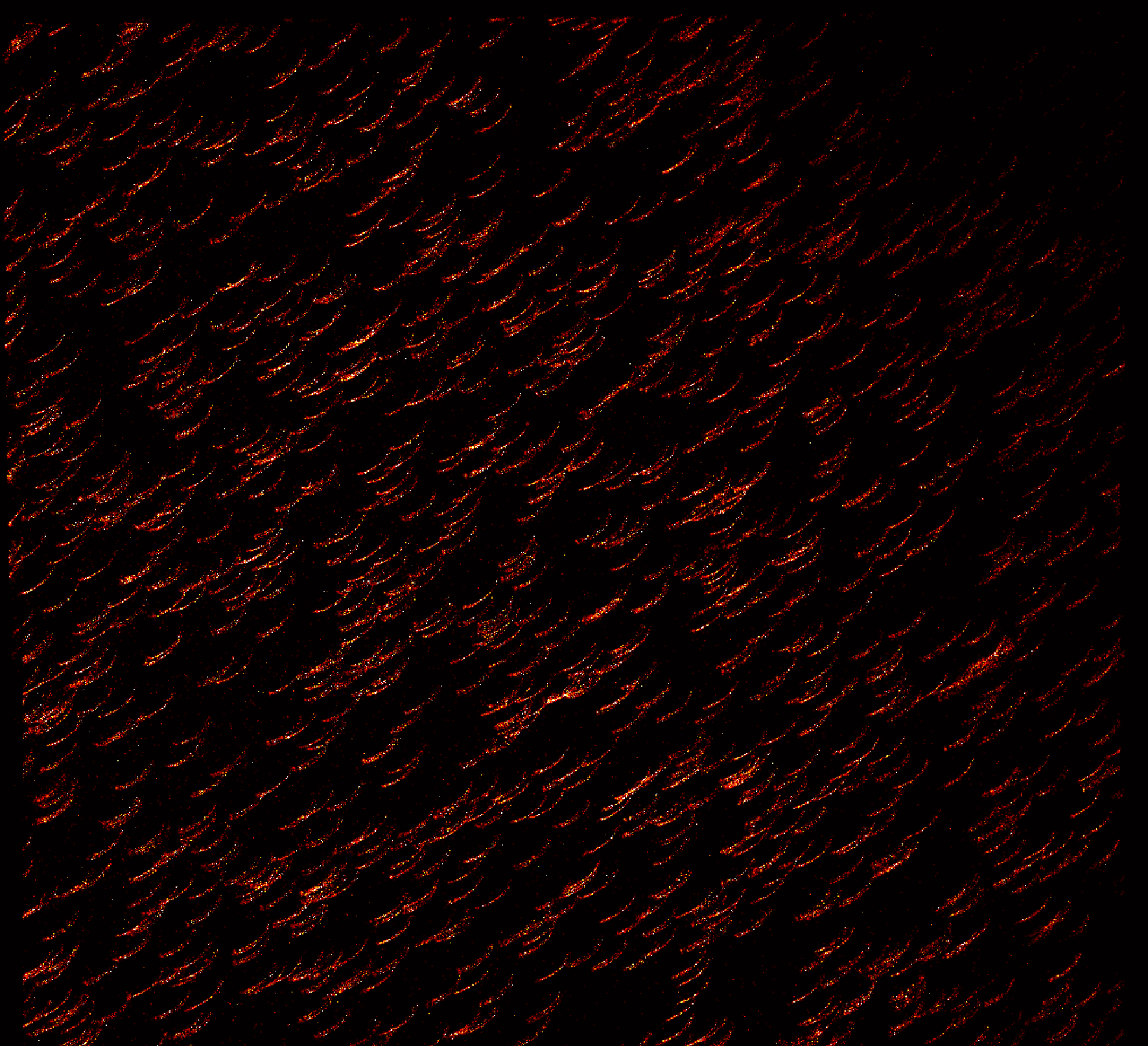

Supplement: Supplementary file 2 — Supplementary Software. [file 41598_2021_2850_MOESM2_ESM.zip › SupplementarySoftware/ExpectedRESULTS/DNA-PAINT_DriftImage.png]

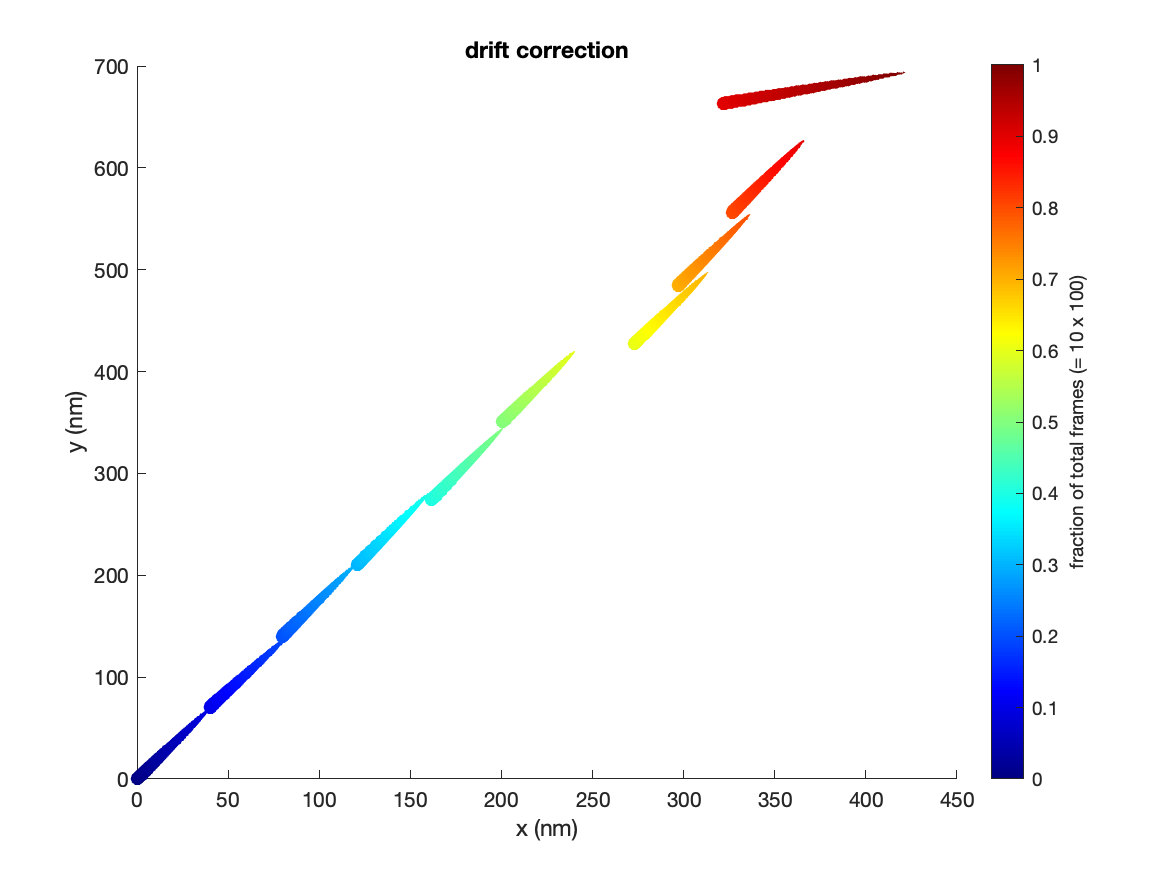

Supplement: Supplementary file 2 — Supplementary Software. [file 41598_2021_2850_MOESM2_ESM.zip › SupplementarySoftware/ExpectedRESULTS/2Dsim_DC.png]

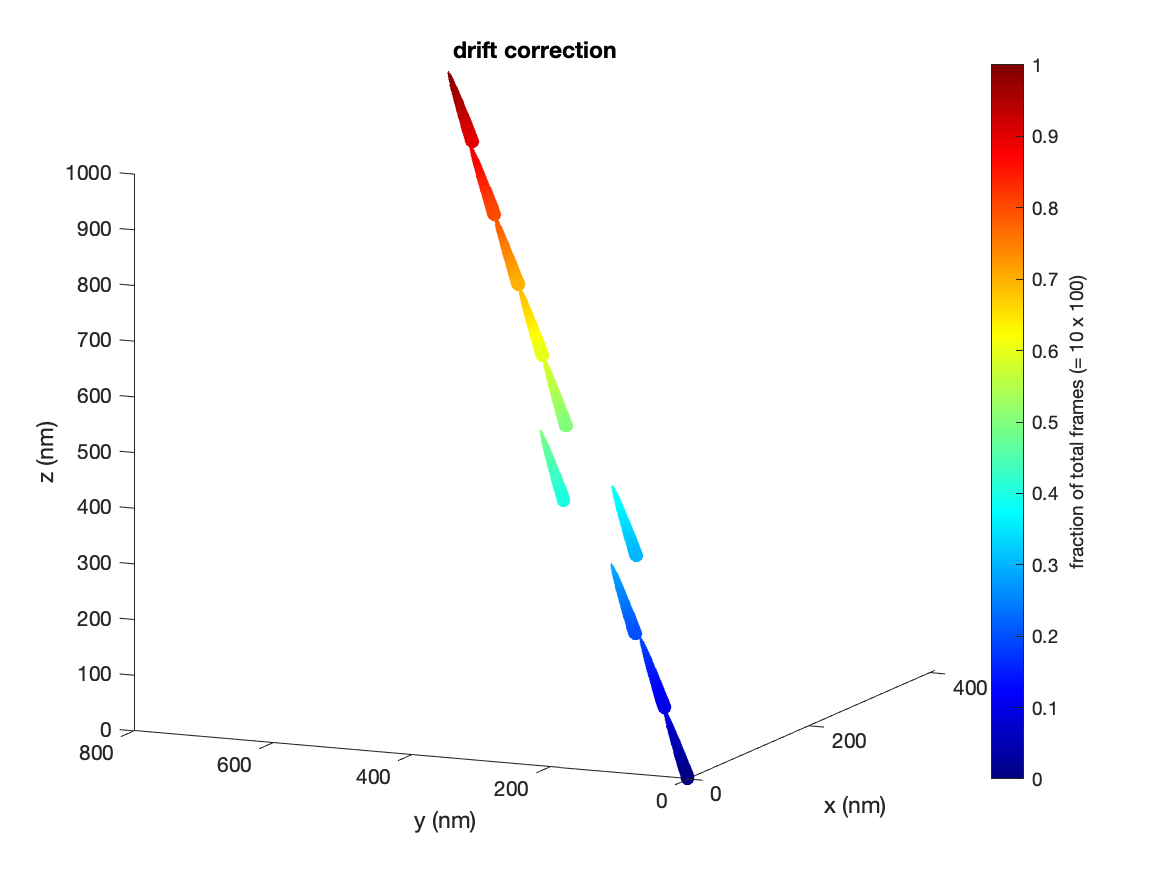

Supplement: Supplementary file 2 — Supplementary Software. [file 41598_2021_2850_MOESM2_ESM.zip › SupplementarySoftware/ExpectedRESULTS/3Dsim_DC.png]

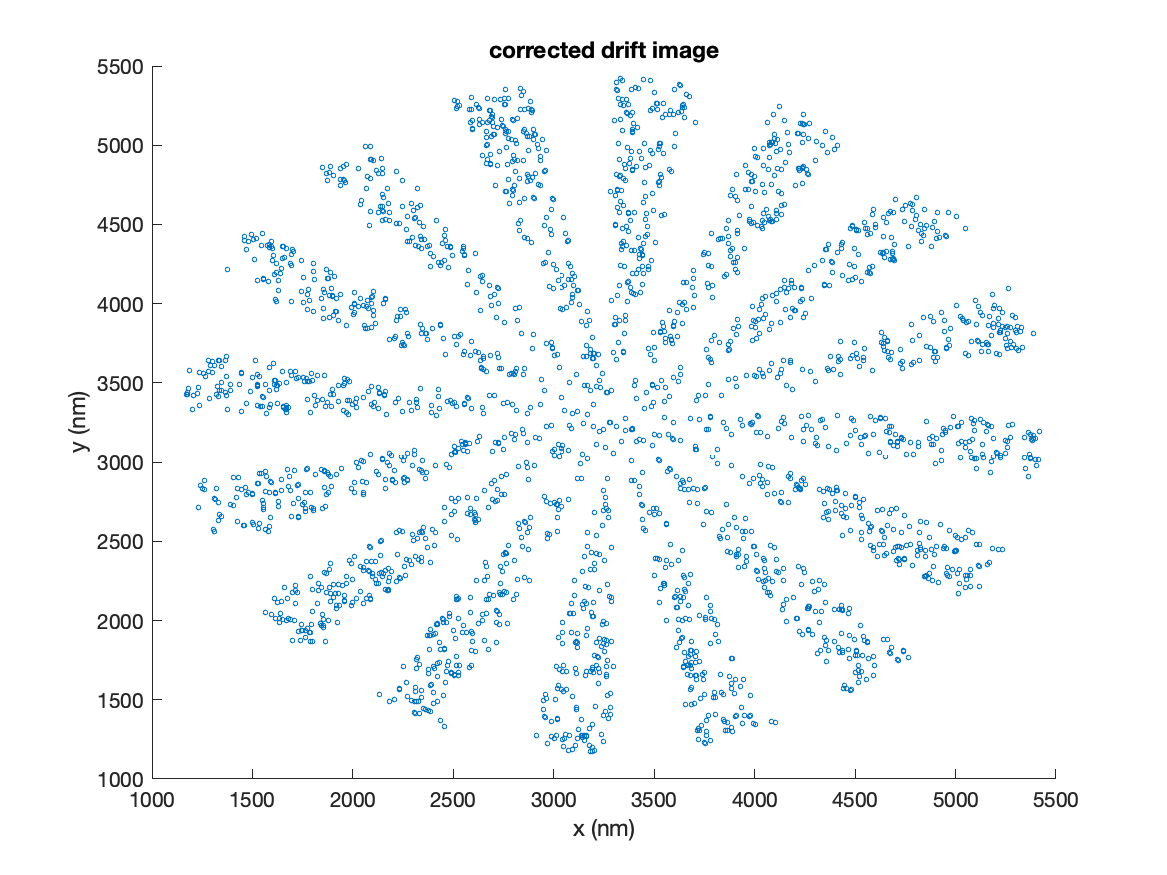

Supplement: Supplementary file 2 — Supplementary Software. [file 41598_2021_2850_MOESM2_ESM.zip › SupplementarySoftware/ExpectedRESULTS/2Dsim_correctedDriftImage.png]

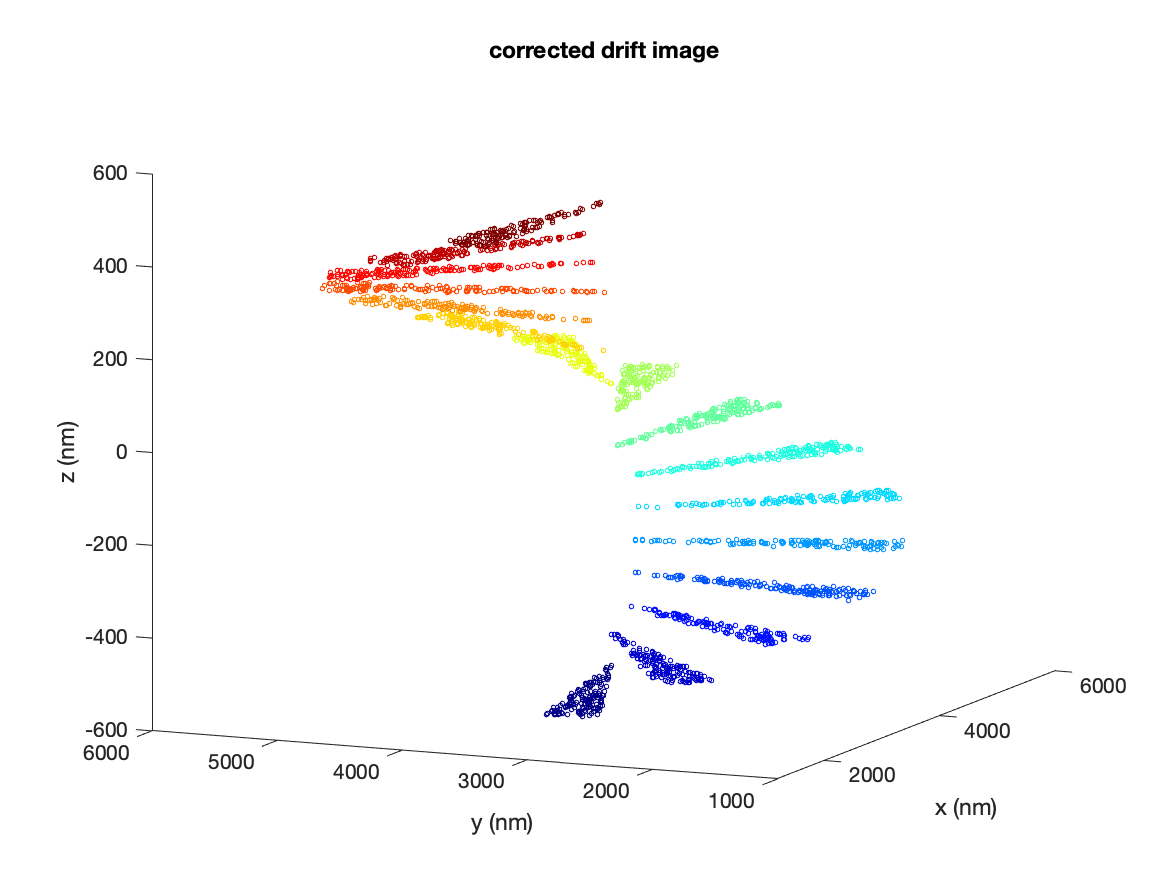

Supplement: Supplementary file 2 — Supplementary Software. [file 41598_2021_2850_MOESM2_ESM.zip › SupplementarySoftware/ExpectedRESULTS/3Dsim_correctedDriftImage.png]

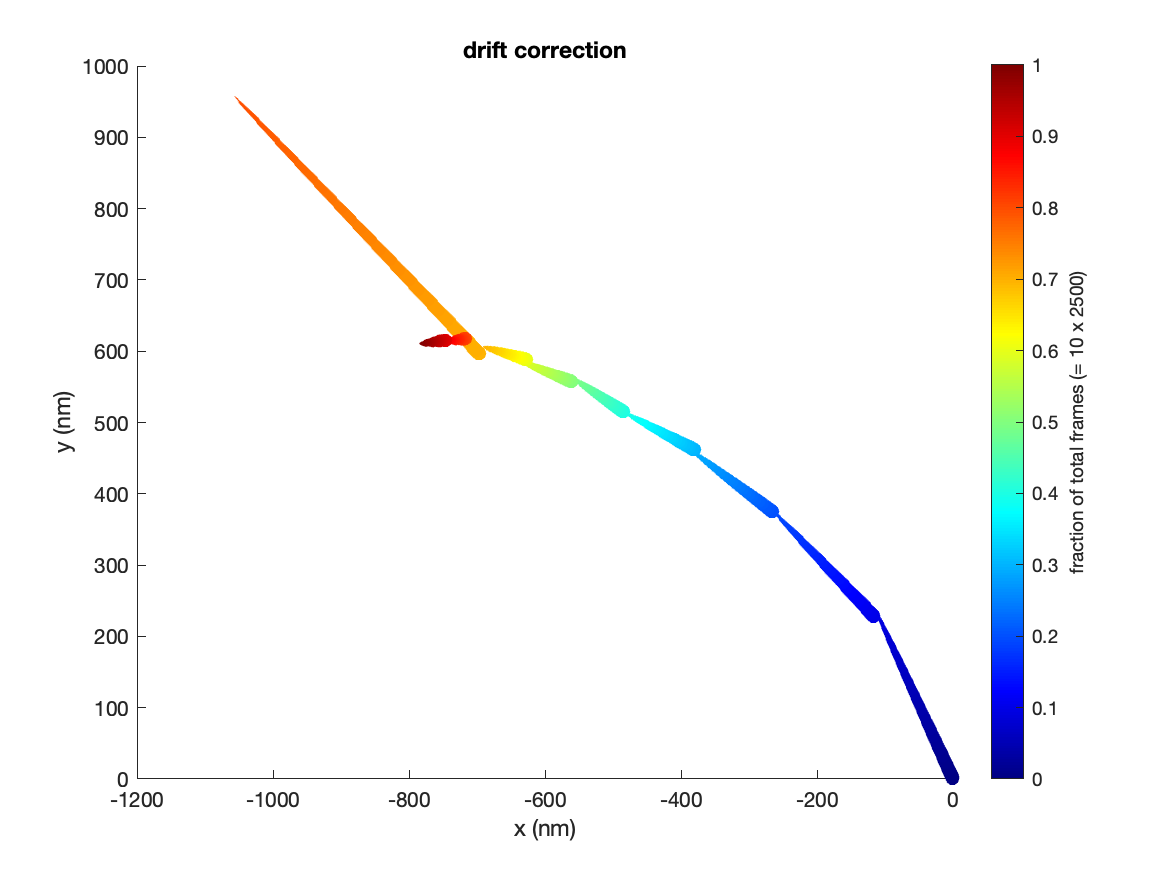

Supplement: Supplementary file 2 — Supplementary Software. [file 41598_2021_2850_MOESM2_ESM.zip › SupplementarySoftware/ExpectedRESULTS/DNA-PAINT_DC.png]

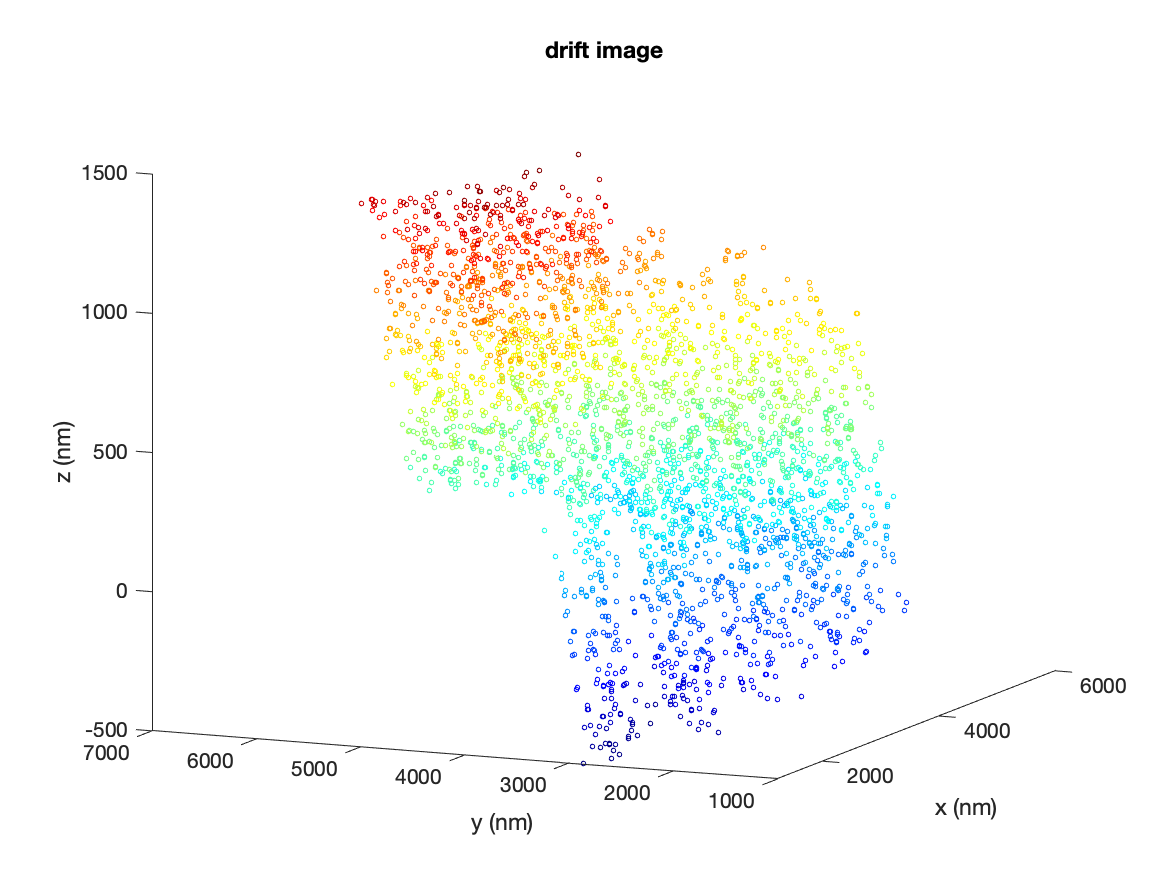

Supplement: Supplementary file 2 — Supplementary Software. [file 41598_2021_2850_MOESM2_ESM.zip › SupplementarySoftware/ExpectedRESULTS/3Dsim_DriftImage.png]

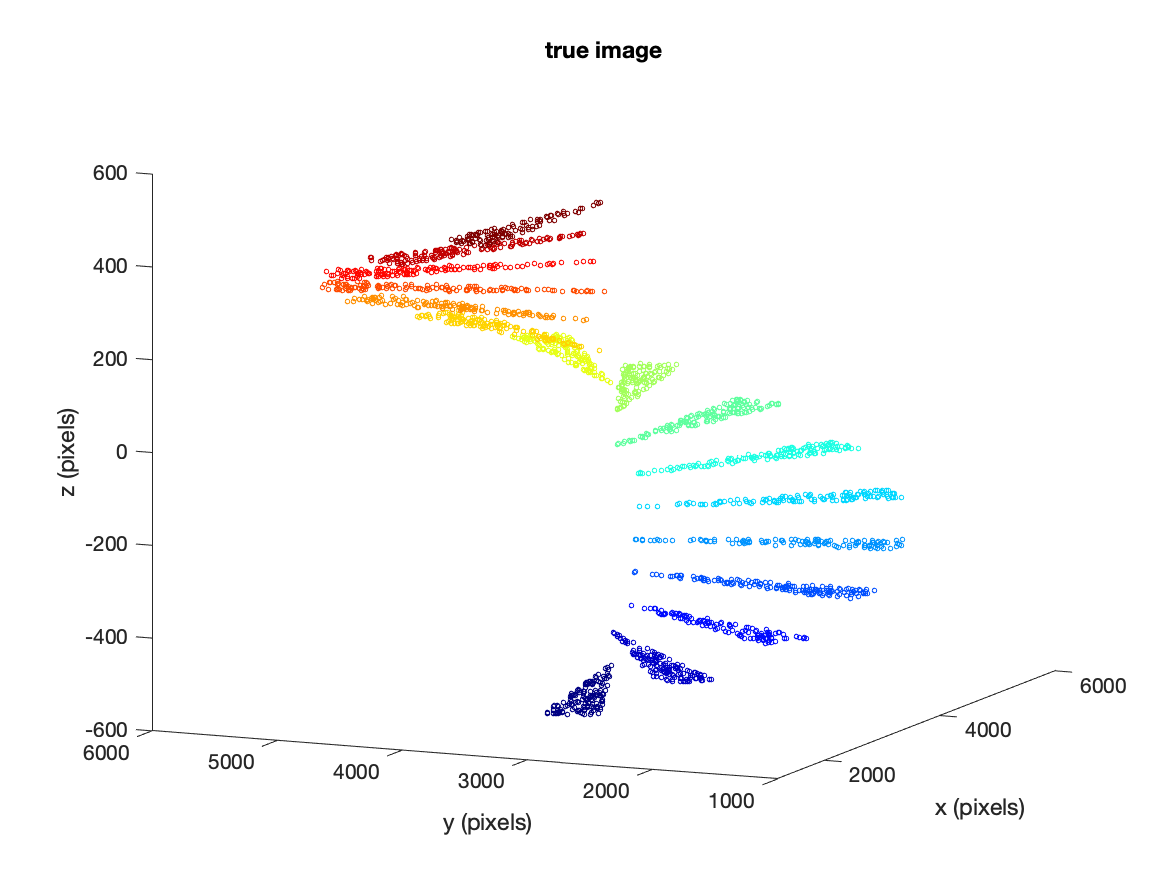

Supplement: Supplementary file 2 — Supplementary Software. [file 41598_2021_2850_MOESM2_ESM.zip › SupplementarySoftware/ExpectedRESULTS/3Dsim_TrueImage.png]

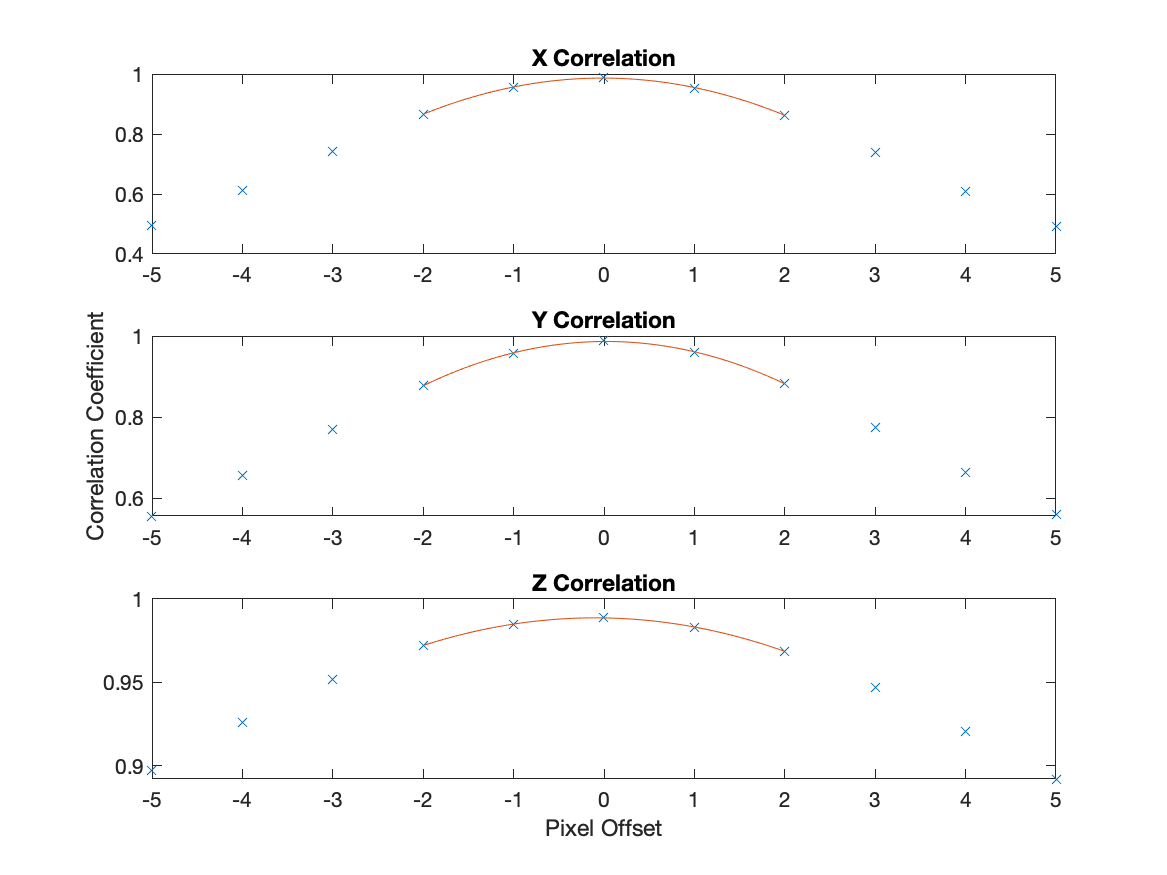

Supplement: Supplementary file 2 — Supplementary Software. [file 41598_2021_2850_MOESM2_ESM.zip › SupplementarySoftware/ExpectedRESULTS/Reg3DTrans_Corr.png]

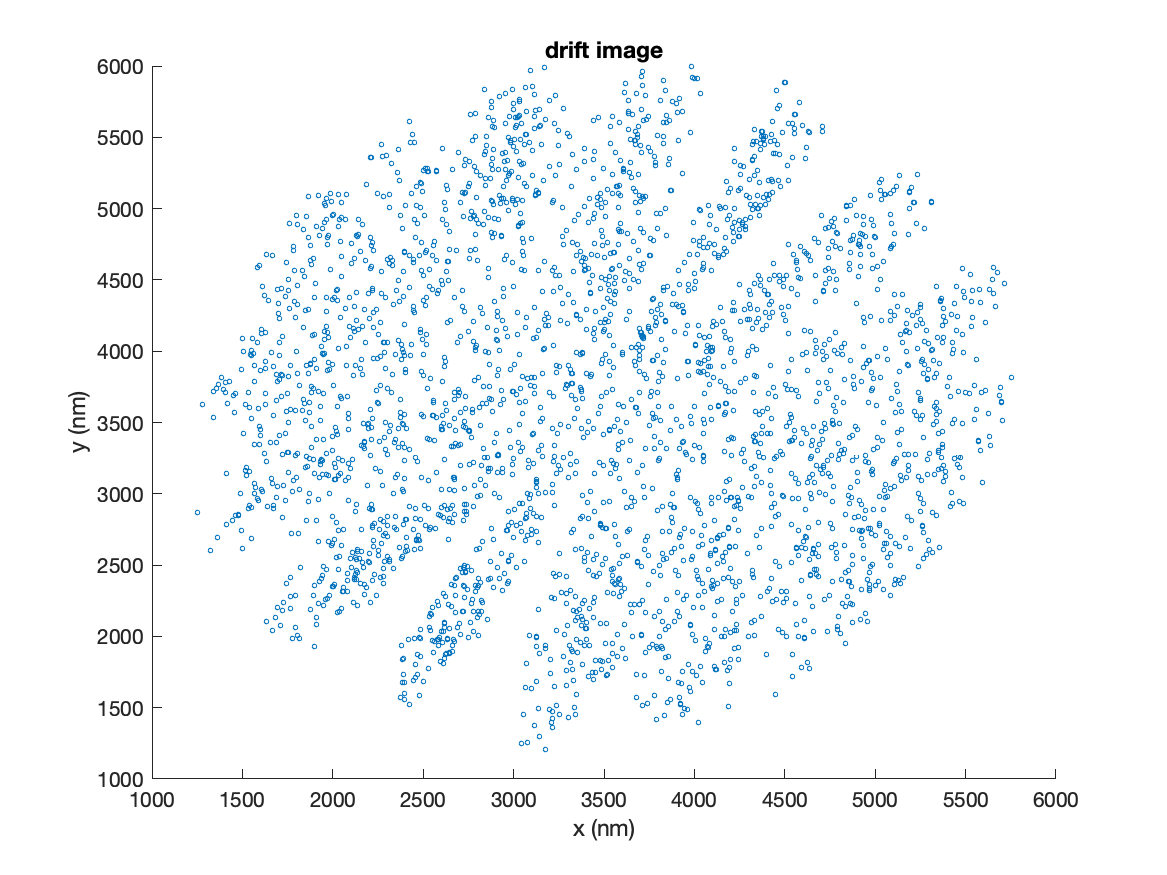

Supplement: Supplementary file 2 — Supplementary Software. [file 41598_2021_2850_MOESM2_ESM.zip › SupplementarySoftware/ExpectedRESULTS/2Dsim_DriftImage.png]

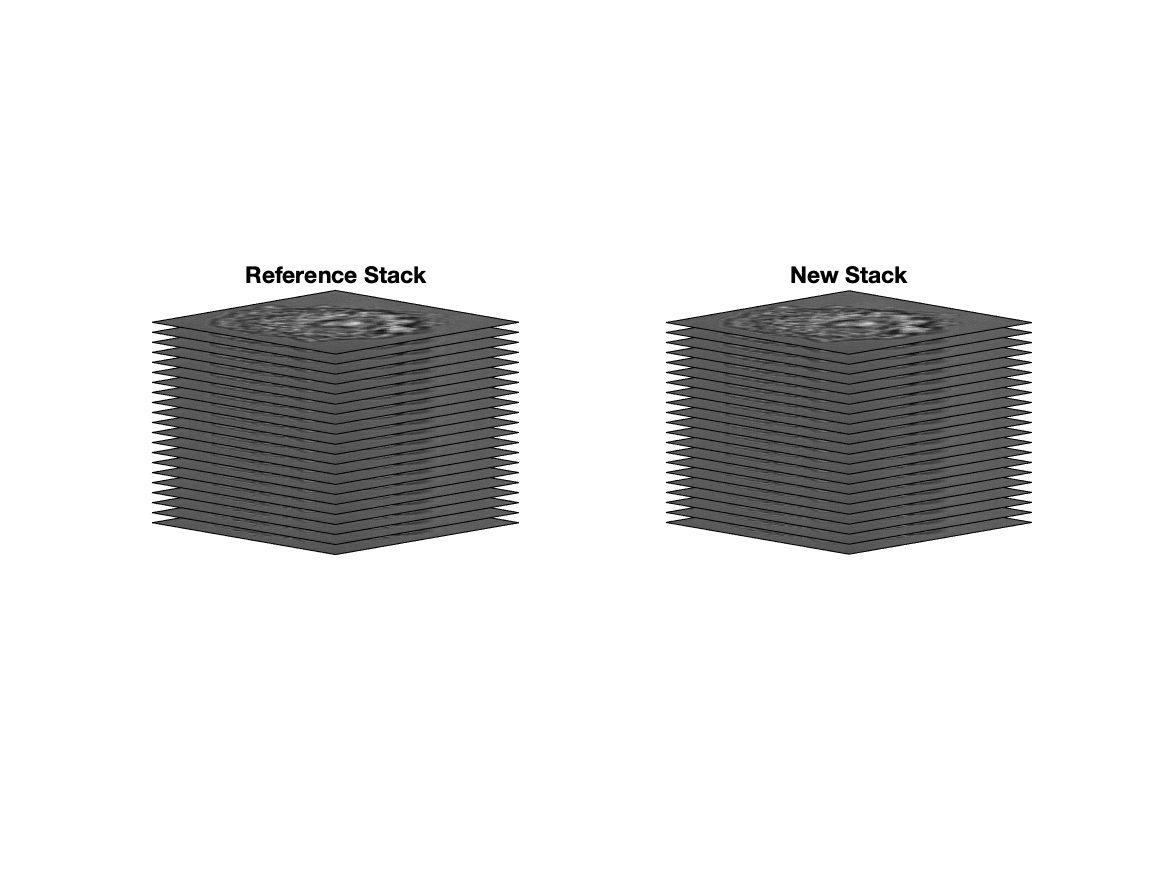

Supplement: Supplementary file 2 — Supplementary Software. [file 41598_2021_2850_MOESM2_ESM.zip › SupplementarySoftware/ExpectedRESULTS/Reg3DTrans_Stacks.png]

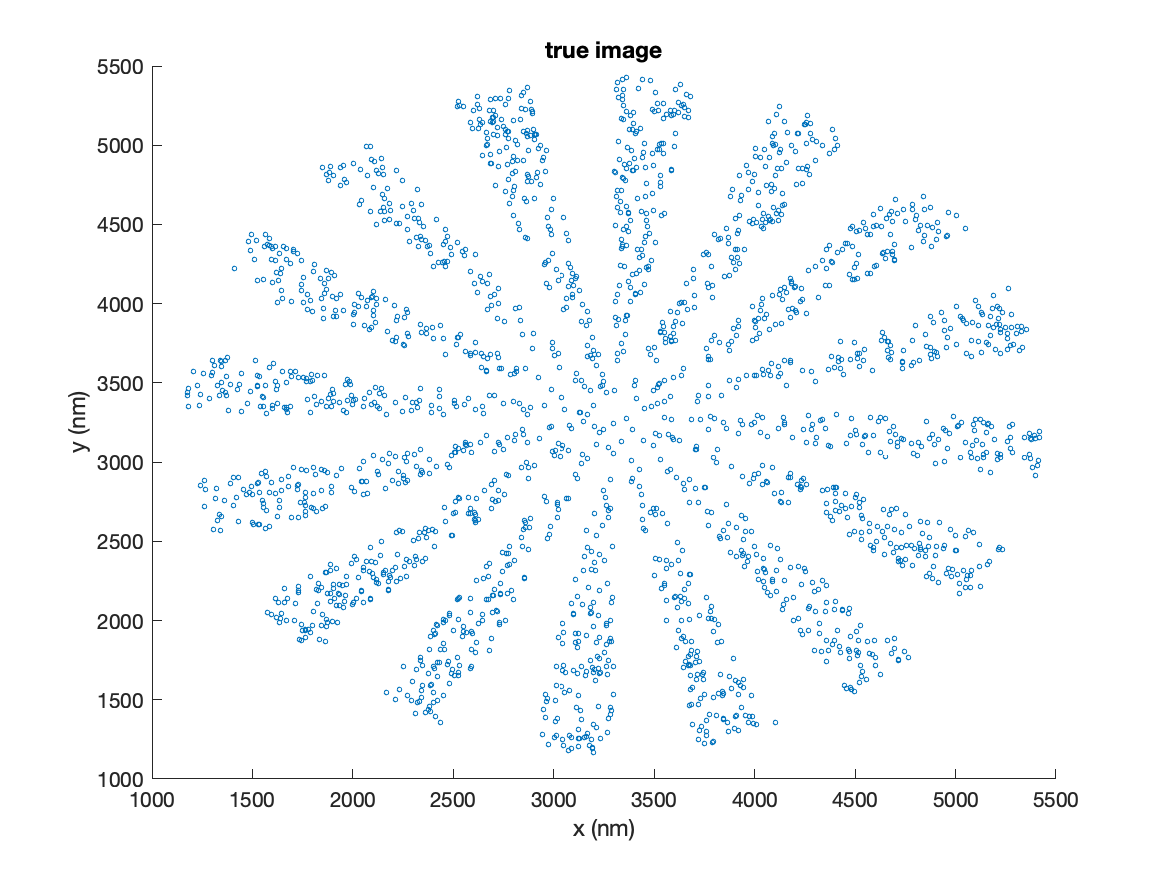

Supplement: Supplementary file 2 — Supplementary Software. [file 41598_2021_2850_MOESM2_ESM.zip › SupplementarySoftware/ExpectedRESULTS/2Dsim_TrueImage.png]

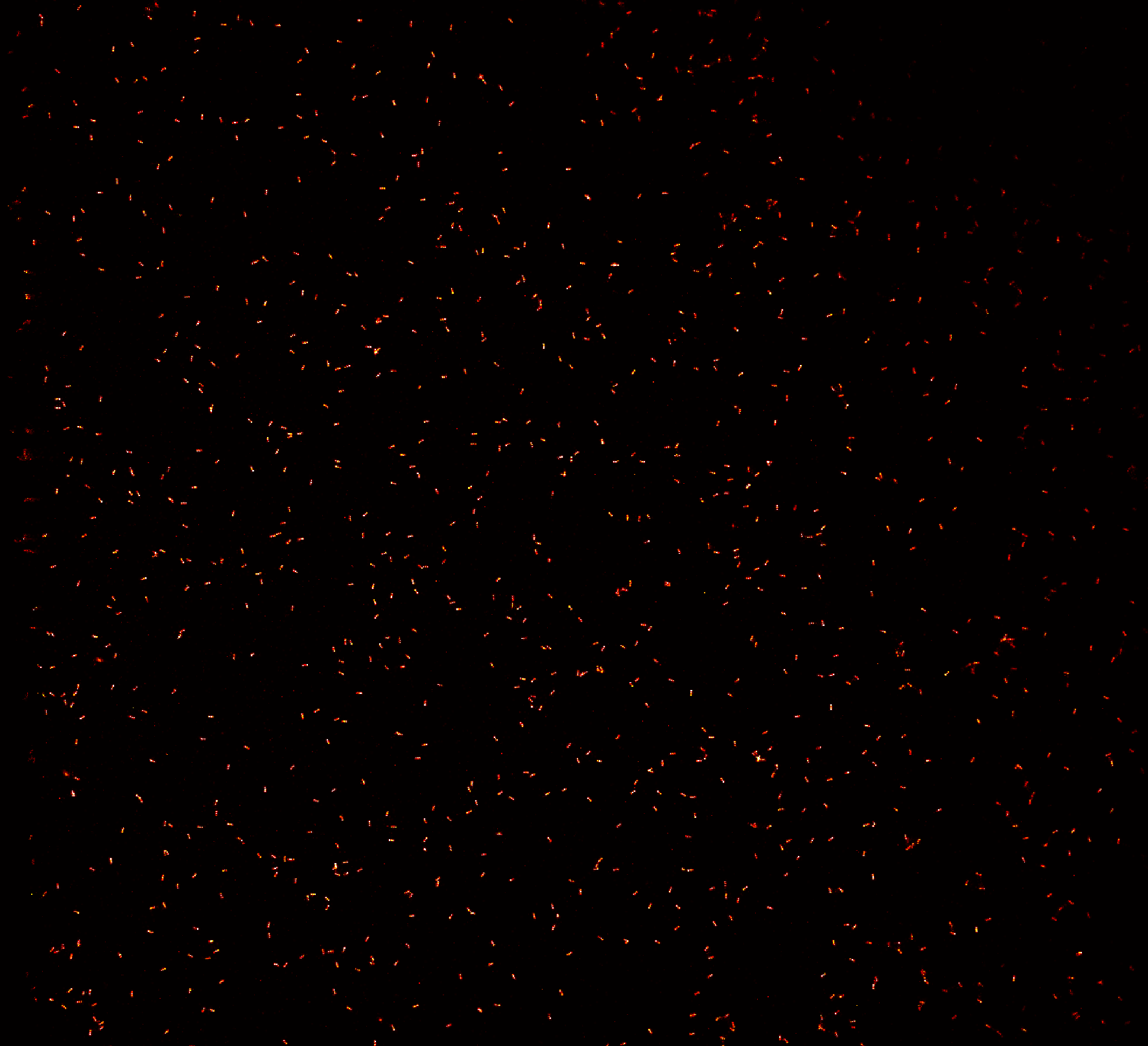

Supplement: Supplementary file 2 — Supplementary Software. [file 41598_2021_2850_MOESM2_ESM.zip › SupplementarySoftware/ExpectedRESULTS/DNA-PAINT_correctedDriftImage.png]
